# Identification of the niche and mobilization mechanism for tissue-protective multipotential bone marrow ILC progenitors

**DOI:** 10.1126/sciadv.abq1551

**Published:** 2022-11-23

**Authors:** Qingyang Liu, Jun Hee Lee, Hyun Min Kang, Chang H. Kim

**Affiliations:** ^1^ Department of Pathology, University of Michigan School of Medicine, Ann Arbor, MI 48109, USA.; ^2^ Mary H. Weiser Food Allergy Center, University of Michigan School of Medicine, Ann Arbor, MI 48109, USA.; ^3^ Immunology Graduate Program, University of Michigan, Ann Arbor, MI 48109, USA.; ^4^ Department of Molecular and Integrative Physiology, University of Michigan Medical School, Ann Arbor, MI 48109, USA.; ^5^ Department of Biostatistics, University of Michigan School of Public Health, Ann Arbor, MI 48109, USA.; ^6^ Rogel Cancer Center, University of Michigan School of Medicine, Ann Arbor, MI 48109, USA.

## Abstract

Innate lymphoid cells (ILCs) play crucial roles in maintenance and defense of peripheral tissues but would undergo natural and inflammation-induced attrition over time. A potential solution to counteract the peripheral ILC attrition would be regulated mobilization of bone marrow (BM) ILC progenitors. The major multipotential ILC progenitors (ILCPs) are divided into two subsets in distinct niches of the BM. Sinusoid ILCPs emigrate from the BM to circulate the peripheral blood. In contrast, parenchyma ILCPs are more likely in cell cycling and less likely to emigrate BM. The mobilization of BM ILCPs is internally and externally controlled by the coordinated expression of the BM retention receptors (Itg-α4 and CXCR4) and the emigration receptors sphingosine-1-phosphate (S1P) receptors. The expression of the BM retention and emigration receptors is developmentally regulated in the steady state and by the inflammasome-derived IL-18. Upon infusion, sinusoid ILCPs can effectively restore peripheral ILC insufficiency and tissue integrity during inflammatory responses.

## Teaser

Tissue-protective multipotential ILC progenitors are mobilized from the bone marrow sinusoid niche.

## INTRODUCTION

Innate lymphoid cells (ILCs)—composed of group 1, 2, and 3 ILCs (ILC1, ILC2, and ILC3)—play essential roles in protecting tissues from infection, inflammation, and injuries (*1*–*7*). ILCs are generated from multipotential ILC progenitors, including innate lymphoid cell progenitors (ILCPs) and single potential progenitors [ILC1 precursor (ILC1P), natural killer precursor (NKP), and ILC2P] (*8*–*16*). It has been observed that all major subsets of ILCs in peripheral tissues undergo age-dependent and effector T cell–like attrition over time (*17*, *18*). This creates tissue ILC insufficiency, resulting in weakened innate immunity and compromised barrier tissue integrity. Best examples for such conditions to cause tissue ILC attrition are the inflammatory conditions in colitis and graft-versus-host disease (*17*–*19*).

ILC progenitors and progenitor-like cells are found not only in the bone marrow (BM) but also in peripheral tissues (*20*). The origin and time of influx of these ILC progenitors into peripheral tissues are currently unclear, but it is thought that mobilization of central ILC progenitors from the BM would be important to sustain peripheral ILC progenitors and ILC activity throughout life.

Tissue-homing receptors—such as CCR9, α4β7, and CCR7—are required for optimal ILC population in peripheral tissues such as the intestine (*21*–*23*). However, mature ILCs in peripheral tissues appear largely sessile (i.e., tissue resident) in the steady state compared with T cells (*24*, *25*). This emphasizes the importance of ILC distribution at the progenitor level to maintain the peripheral ILC compartment. We investigated the niches and mobilization mechanisms for BM ILC progenitor populations with the focus on the multipotential ILCPs. We report that a subset of ILCPs, localized in BM sinusoids, is actively mobilized in steady state and inflammatory conditions. The emigration of BM ILCPs is regulated by coordinated expression of the receptors for adhesion and chemotaxis that control their localization, retention, and/or emigration. Moreover, we identified a novel inflammatory cue [i.e., interleukin-18 (IL-18)] that mobilizes BM ILCPs and determined the function of the BM-emigrating ILCPs in restoring intestinal inflammation and injury.

## RESULTS

### Dynamic changes in the population of ILC progenitors in the blood and peripheral tissues

**Fig. 1.**
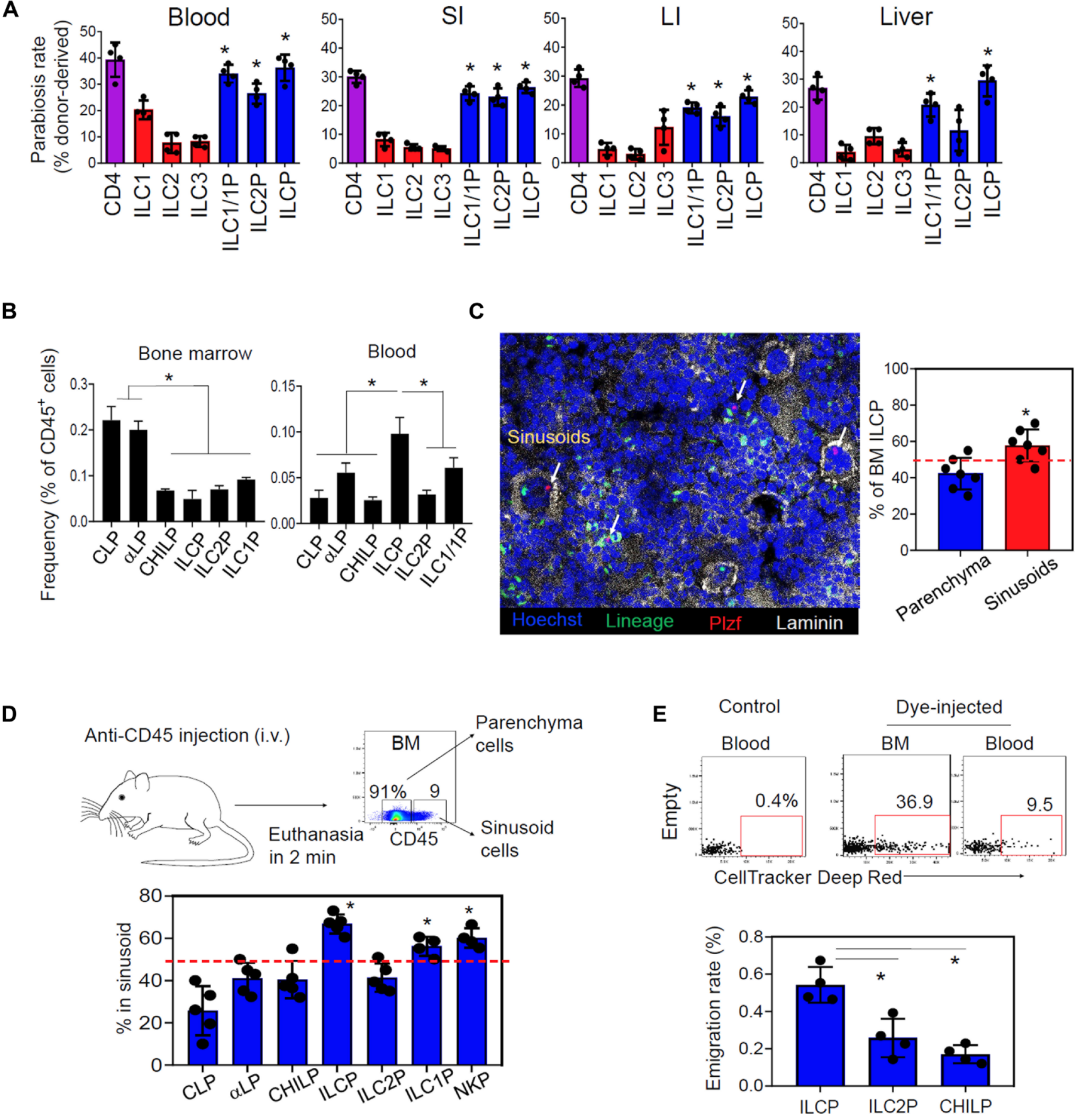
ILCPs are the most actively emigrating ILC progenitors from the BM in steady state. (**A**) Parabiosis between congenic CD45.1 and CD45.2 mice was maintained for 2 months and examined for relative population of host versus donor-derived mature ILCs and ILC progenitor subsets in various tissues. (**B**) Frequency of ILC progenitor subsets in the BM and peripheral blood. (**C**) Immunofluorescent staining of BM sinusoid Lin^−^ PLZF^+^ ILCPs. BM sections were stained to visualize ILCPs. (**D**) Sinusoid cell labeling by intravenous (i.v.) injection of AmCyan-labeled αCD45. Mice were euthanized 2 min later, and % in sinusoid cells was calculated on the basis of the relative frequencies of AmCyan-labeled sinusoid and nonlabeled parenchyma ILC progenitor subsets. (**E**) Frequency of emigrated CellTracker Deep Red–labeled ILC progenitors in the BM and peripheral blood. The CellTracker was injected into the bones of mice before they were euthanized 24 hours later. Pooled data obtained from at least three different experiments (*n* = 4 to 7) are shown. *P < 0.05.

ILC progenitors are present in the blood circulation and peripheral tissues (*20*, *26*–*28*). To understand the dynamic of ILC progenitor populations in the blood and peripheral tissues, we performed a parabiosis study (Fig. 1A). The definitions and cellular features of ILCP, ILC1P, and other progenitors are described in fig. S1 (A and B) and table S1. We verified that Lin^−^CD127^+^CD117^+^PLZF^+^ ILCPs in both the BM and periphery (spleen) had the potential to become ILC1, ILC2, and ILC3 in coculture with OP9-DL1. The Lin^−^CD127^+^NKp46^+^NK1.1^+^CD49a^+^ cells in the BM had the potential to mainly become ILC1 and did not include mature T-bet^+^ ILC1 (fig. S1). This identification method, however, can include ILC1 and, therefore, is not specific for ILC1P in the periphery (table S1). Thus, we call these cells ILC1P/ILC1. In parabiosis mice, we found that ILC progenitors (ILCP, ILC1P/ILC1, and ILC2P) in the blood and most peripheral tissues—such as the spleen, lymph nodes, liver, lung, small intestine (SI), and large intestine (LI)—had unexpectedly high turnover rates (Fig. 1A and fig. S2, A and B). The replacement rates for the blood and tissue ILC progenitors were similar to those of CD4^+^ T cells, which are largely recirculating lymphocytes. Despite variations, ILCPs displayed the highest levels of replacement rates among the examined ILC progenitors. In contrast to and in line with a previous observation (*24*), mature ILCs in most peripheral tissues underwent low levels of replacement except the ILC1 in the blood (Fig. 1A and fig. S2, A and B). Thus, ILC progenitors appear highly dynamic in tissue population unlike their mature counterparts.

### Distinct niches and emigration behaviors of BM ILC progenitor populations

To identify the major circulating ILC progenitors, we compared the frequency of ILCPs, ILC1P/ILC1, and ILC2P in the blood versus BM (Fig. 1B and fig. S2A). ILCPs, while they formed a relatively small population in the BM, were the most abundant circulating ILC progenitors (Fig. 1B and fig. S3A).

The mammalian BM microenvironment is divided into distinct niches. Hematopoietic stem and progenitor cells are found in the endosteal and central niches, and the central niche is further divided into subniches around arterioles and sinusoids (*29*, *30*). We performed confocal microscopy and found that the majority of promyelocytic leukemia zinc finger–positive (PLZF^+^) ILCPs are localized within and around the sinusoid niche (Fig. 1C). Few PLZF^+^ ILCPs were found in the endosteal niche close to the bone (fig. S3B). We took an antibody labeling technique (*31*) to further verify the presence of ILCPs in the sinusoid niche. We administered intravenous AmCyan-tagged anti-CD45 antibody into mice and identified AmCyan-labeled (sinusoid) and nonlabeled (parenchyma, including nonsinusoid central and endosteal niches) cells in the BM. This approach assigned ~70% ILCPs and ~60% ILC1P and NKP to the sinusoidal niche (Fig. 1D and fig. S3C). In contrast, ~40% of ILC2P were assigned to the sinusoidal niche. These data indicate that ILC progenitor populations are heterogeneous in their localization within the BM, and the majority of the multipotential ILCPs are present in the sinusoid niche, which is considered not only a major site of hematopoiesis but also the gate for cell trafficking in and out of the BM (*32*). To determine whether these BM ILC progenitors emigrate to the peripheral blood, we took a BM CellTracker microinjection approach (*33*). Emigration rates were calculated on the basis of the ratio of labeled cells in the blood to those remaining in the BM for each ILC progenitor subset. The emigration rate of ILCPs was significantly higher than that of ILC2P and common helper ILC precursor (CHILP) (Fig. 1E and fig. S3D). Thus, the ILCP population is highly active in emigration from the BM in the steady state. Hereafter, we will call the ILCP subsets in the BM parenchyma and sinusoid niches “pILCPs” and “sILCPs,” respectively.

### Molecular features of pILCPs versus sILCPs for differentiation and emigration

**Fig. 2.**
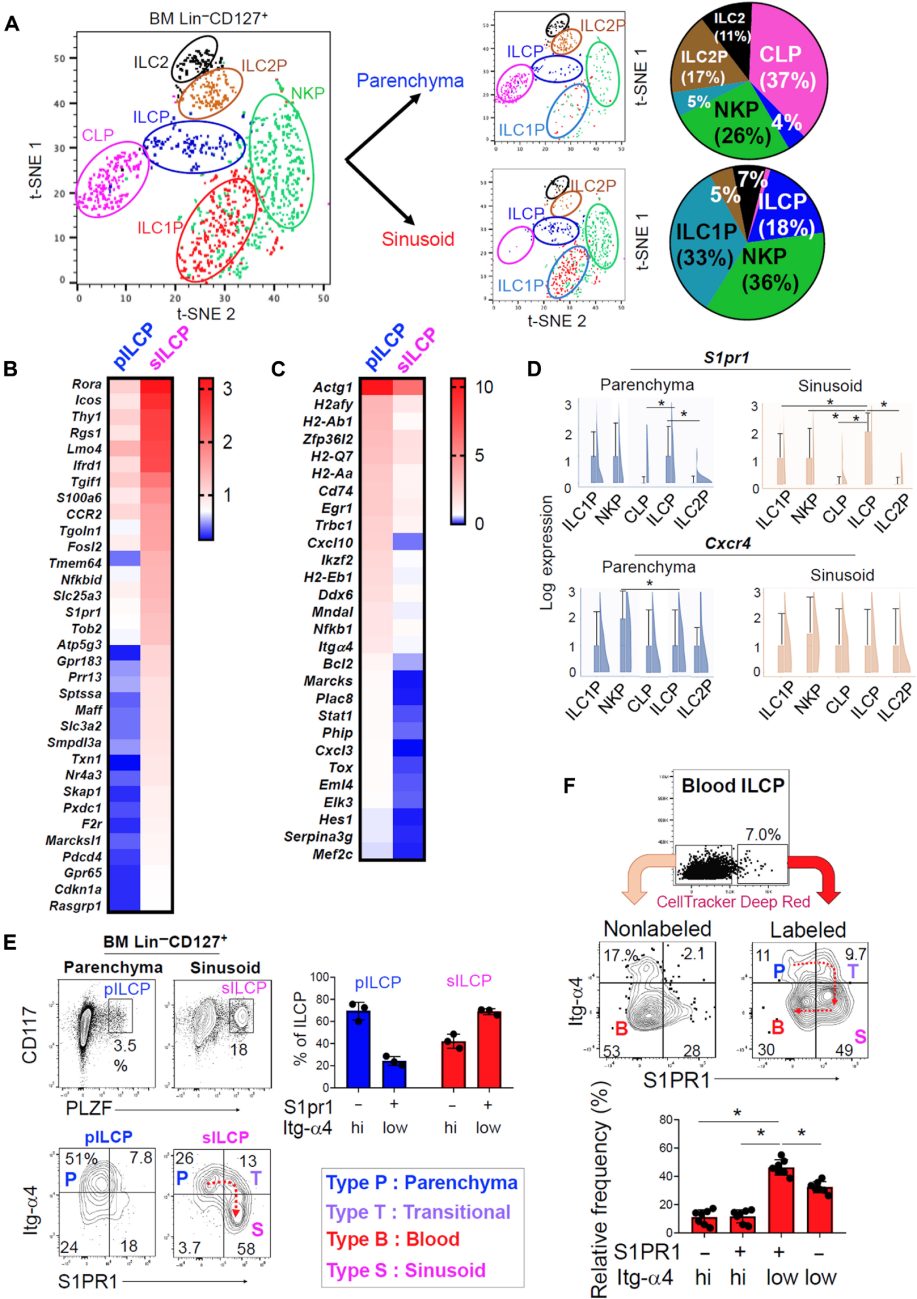
The two populations of ILCPs in the BM parenchyma or sinusoid niches have distinctive expression of BM retention and emigration receptors. (**A**) scRNA-seq analysis of BM pILCP versus sILCP. Unbiased t-SNE clustering is shown for BM parenchyma and sinusoid ILCPs. Composition of ILC progenitors identified by the t-SNE plots in the two compartments of the BM is shown. (**B**) A heatmap for up-regulated (≥2-fold) genes in sILCP. (**C**) A heatmap for down-regulated (≥2-fold) genes in sILCP. (**D**) Violin and box plots for the expression of *S1pr1* and *Cxcr4* on parenchyma versus sinusoid ILC progenitor subsets. (**E**) Coexpression of Itg-α4 and S1PR1 on pILCP and sILCP. (**F**) Expression of Itg-α4 and S1PR1 on recently emigrated BM ILCPs versus circulating ILCPs in the peripheral blood. Pooled data obtained from three different experiments (*n* = 3 to 6) are shown. *P < 0.05.

To gain insights into the molecular features of the pILCP and sILCP subsets, we performed a single-cell RNA sequencing (scRNA-seq) analysis for the sinusoid (anti-CD45/AmCyan labeled) and parenchyma (nonlabeled) Lin^−^CD127^+^ BM cells. T-distributed stochastic neighbor embedding (t-SNE) dimensional reduction analysis of the single-cell transcriptome identified common lymphoid progenitors (CLPs), ILCPs, ILC1P, NKP, ILC2P, and ILC2 (Fig. 2A and fig. S4, A and B). ILCP, while a minority in the BM, was the third largest population in the sinusoid after NKP and ILC1P. In contrast, ILC2P was underrepresented in the sinusoid compared with the parenchyma compartment (Fig. 2A). A pseudotime trajectory analysis positioned CLP as the starting point and the more differentiated ILC2P/ILC2 and ILC1P/NKP as the end points along the trajectory and placed ILCPs (pILCPs and sILCPs) between the CLP and the single potential progenitor populations (fig. S4C).

Top genes that were more highly expressed in sILCPs, compared with pILCPs, included *Rora*, *Icos*, *Thy1*, *Lmo4*, *Ifrd1*, *S100a6*, *CCR2* (a chemokine receptor), *Tgoln1*, *Fosl2*, *Trem64*, *Nfkbid*, *Slc25a3* (mitochondrial phosphate transporter), *S1pr1* [an sphingosine-1-phosphate (S1P) receptor], *Tob2*, and *Gpr183* (an oxysterol receptor) (Fig. 2B) (*34*). *Rora*, *Icos*, *Thy1*, and *Lmo4* are highly expressed by ILC progenitors (*9*, *35*–*37*). RORα and Lmo4 are involved in the ILC lineage commitment (*27*, *35*, *37*). Top genes that were down-regulated in sILCPs compared with pILCPs included *Bcl2*, *Actg1*, several major histocompatibility complex II molecules (*H2afy*, *H2-Ab1*, *H2-Q7*, *H2-Aa*, and *H2-Eb1*), *Cd74*, *Egr1*, *Nfkb1*, and *Itga4*, among others (Fig. 2C). Pathway analysis based on the transcriptomic differences predicted that the two ILCP subsets would differ in localization, locomotion, anatomical structure, and metabolic and developmental processes (fig. S5, A and B). BM Lin^−^ CD127^+^ CD117^+^ PLZF^+^ ILCPs are largely GATA3^+^ as previously reported (*10*). Only a small subset (~5%) of these cells expressed retinoid orphan receptor gamma t (RORγt) (fig. S6, A and B). In peripheral tissues, Lin^−^ CD127^+^ CD117^+^ PLZF^+^ cells lost the GATA3 expression, but small subsets of these cells expressed T-bet or RORγt. While rare among ILC lineage cells in the BM, ~60% of RORγt^+^ cells were found in the sinusoid compartment, and ~60% of these sinusoid RORγt^+^ cells expressed CCR6 (fig. S7, A to C). We also verified that the expression of Inducible T Cell Costimulator (ICOS) and Inhibitor of DNA binding 2 (ID2) was increased in sinusoid ILCPs at protein level (fig. S7D).

A key feature of sILCPs is their relatively high expression of *S1pr1* compared with pILCPs and committed progenitors such as NKP, ILC1P, and ILC2P (Fig. 2D). However, there was no difference between sILCPs and pILCPs in the expression of *Cxcr4* mRNA and CD69 (Fig. 2D and fig. S8A). The integrin (Itg)–α4β1 is a key cell adhesion receptor for vascular cell adhesion molecule–1 (VCAM-1) and fibronectin, which are expressed by stromal cells and in extracellular matrix (ECM) for hematopoiesis and cell retention in the BM (*38*). Most BM ILCPs uniformly expressed α4β7 and Itg-β1 (fig. S8, B and C), but they were composed of Itg-α4^high (hi)^ Itg-β1^+^ and Itg-α4^low^ Itg-β1^+^ cells. While most pILCPs were Itg-α4^hi^S1PR1^−^ (type “P” for parenchyma), sILCPs included Itg-α4^low^S1PR1^+^ cells as its major subset (type “S” for sinusoid) along with the minor Itg-α4^hi^S1PR1^−^ (P) and Itg-α4^hi^S1PR1^+^ (type “T” for transitional) subsets (Fig. 2E). In the peripheral blood, ILCPs are predominantly Itg-α4^low^S1PR1^−^ cells (type “B” for blood) (Fig. 2F). Our BM CellTracker microinjection study revealed that type S (Itg-α4^low^S1PR1^+^) ILCPs were most enriched in the BM-emigrating cells. The emigrated ILCPs also included the type B (Itg-α4^low^S1PR1^−^) subset, which appears to have been derived from the Itg-α4^low^S1PR1^+^ subset in the blood after emigration, potentially following S1P-dependent internalization of surface S1PR1. Thus, the ILCPs in the sinusoid niche, equipped with the emigration receptor but down-regulated for the retention receptor, appear to emigrate the BM most efficiently.

### sILCPs efficiently generate committed ILC progenitors and mature ILCs in the periphery

**Fig. 3.**
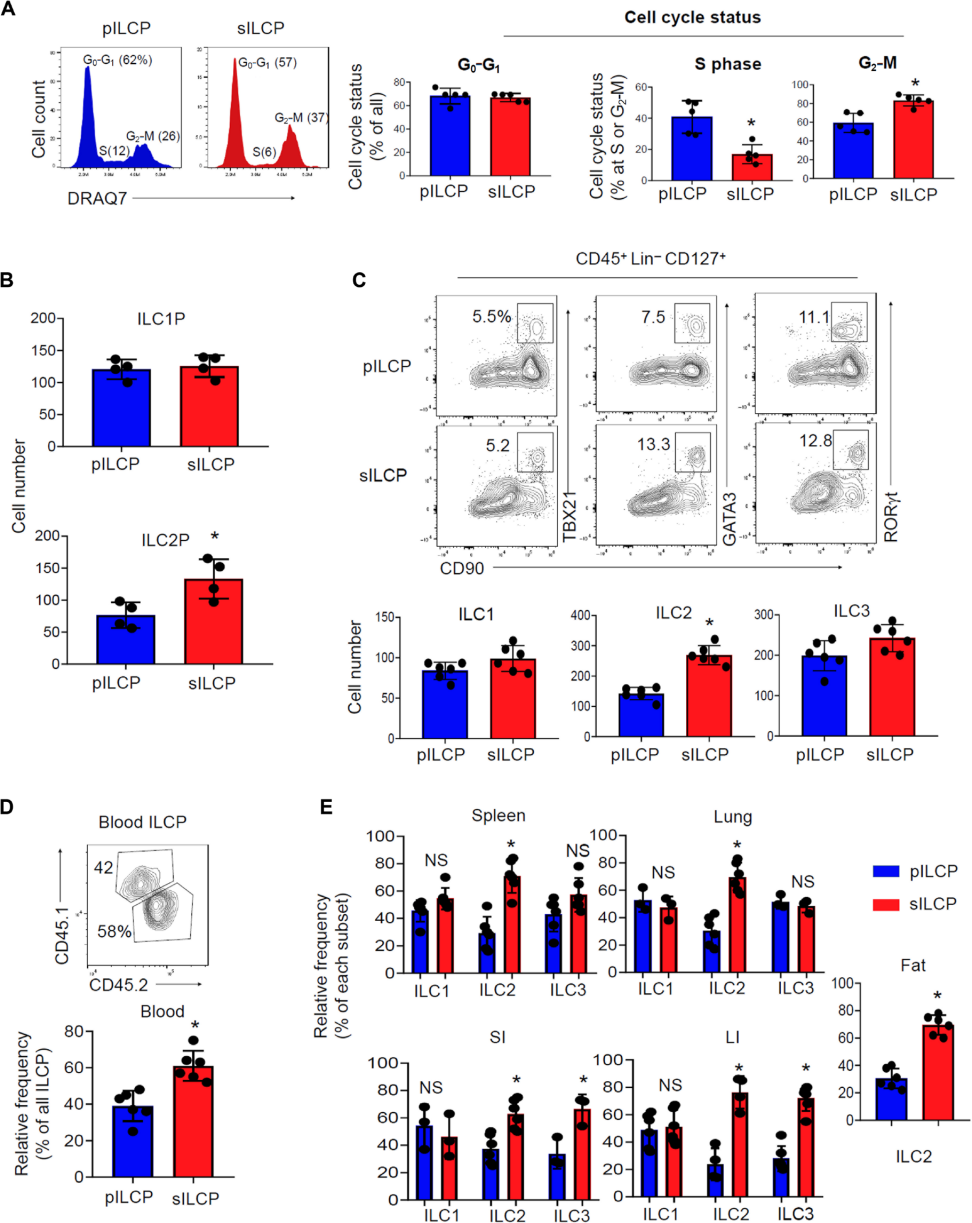
Cell cycle status and differentiation potential of pILCPs and sILCPs. (**A**) Cell cycle status (G_0_-G_1_, S, and G_2_-M) of pILCPs and sILCPs. (**B** and **C**) In vitro differentiation of pILCPs and sILCPs. Sorted pILCPs (AmCyan^−^Lin^−^CD127^+^CD117^+^ PLZF^+^ cells) and sILCPs (AmCyan^+^Lin^−^CD127^+^CD117^+^ PLZF ^+^ cells) following BM sinusoid labeling of PLZF^GFP^ mice with anti-CD45-AmCyan were cultured on OP9-DL1 cells for 10 to 12 days. ILC progenitor subsets and mature ILC subsets were examined by flow cytometry after culture. (**D** and **E**) In vivo differentiation of pILCPs and sILCPs. Sorted sILCPs and pILCPs were cotransferred intravenously into *Rag2*^−/−^*IL2R*γ^−/−^ mice. Mice were euthanized ~4 weeks later, and the relative frequencies of blood ILCPs (D) and tissues ILC subsets (E) were examined. Representative and pooled data obtained from at least three different experiments (*n* = 4 to 6) are shown. *P < 0.05. NS, not significant.

Because of their distinct niches in the BM, we examined the cell cycling status of pILCPs and sILCPs. While both subsets included cells in the S and G_2_-M phases, pILCPs were relatively more in the S phase, whereas relatively more sILCPs were in the G_2_-M phase (Fig. 3A). Because the S phase precedes G_2_-M phase, sILCPs appear in later phases of cell cycling compared with pILCPs, which is relatively more active in DNA synthesis.

We next investigated whether sILCPs and pILCPs have similar or distinct differentiation potentials. We sorted sILCPs and pILCPs and determined their differentiation potential in vitro and in vivo. In culture with OP9-DL1, both sILCPs and pILCPs generated ILC1P and ILC2P, as well as mature ILC1, ILC2, and ILC3 (Fig. 3, B and C). sILCPs made CCR6^−^ ILC3 but few CCR6^+^ LTi ILC3 (fig. S9), which is consistent with the established differentiation potential of ILCPs in general (*10*). While the two populations of ILCPs were overall similar, sILCPs were relatively more efficient in the production of ILC2P and ILC2. For in vivo differentiation, we performed a competitive population study for sILCPs and pILCPs in the lymphocyte-deficient Rag2^−/−^IL2Rg^−/−^mice (Fig. 3, D and E). Many more ILCPs in the blood were derived from sILCPs compared with pILCPs (Fig. 3D). Both sILCPs and pILCPs were able to generate ILC1, ILC2, and ILC3, but sILCPs were more efficient producers of ILC2 in the spleen, lung, SI, LI, and white fat (Fig. 3E). sILCPs were relatively more efficient than pILCPs in producing ILC3 in the intestines. These data indicate that sILCPs can efficiently replenish the peripheral ILC system composed of ILC progenitors and mature ILCs.

### The emigration of ILCPs is reciprocally regulated by the BM and bloodborne chemoattractants

**Fig. 4.**
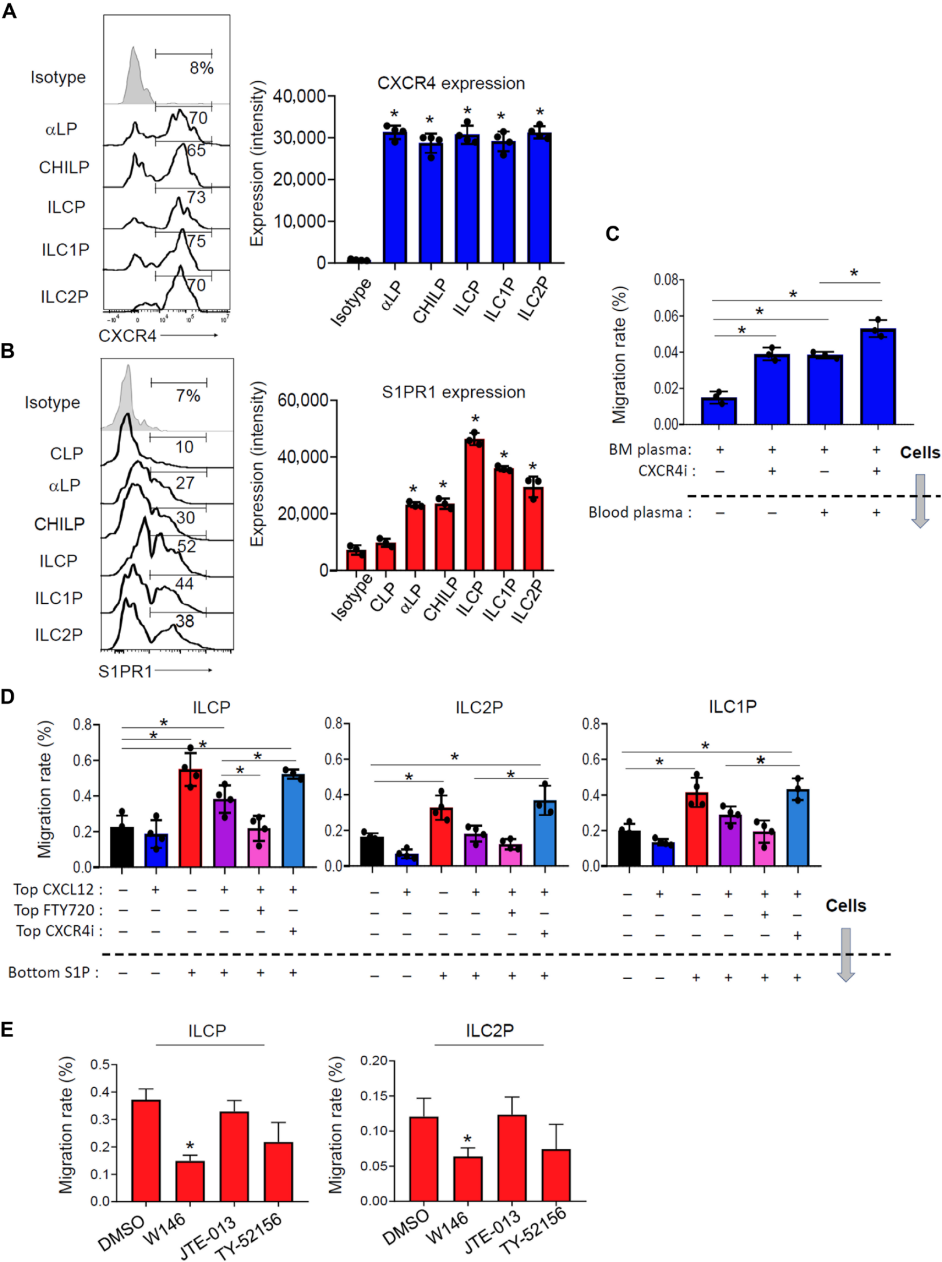
Migratory behavior of ILC progenitors in response to the negative BM and positive bloodborne chemotactic gradients. (**A**) Expression of cell surface CXCR4 by BM ILC progenitor subsets. (**B**) Expression of cell surface S1PR1 by BM ILC progenitors. (**C**) Chemotaxis of BM ILC progenitors to BM fluid and diluted (1:10) plasma. AMD3100 (CXCR4i) was used to block the CXCR4 function. (**D**) Chemotaxis of BM ILC progenitors to CXCL12 (100 ng/ml) in a negative gradient and S1P (100 nM) in a positive gradient. FTY720 and AMD3100 were used to inhibit the activities of S1PRs and CXCR4, respectively. (**E**) The S1PR1-specific antagonist W146 effectively inhibited the ILC progenitor chemotaxis to S1P. Also examined were JTE013 (S1PR2 antagonist) and TY52156 (S1PR3>1 antagonist). Pooled data obtained from at least three different experiments (*n* = 3 to 4) are shown. *P < 0.05. DMSO, dimethyl sulfoxide.

Because CXCL12 and S1P are defining chemoattractants for the BM and peripheral blood, respectively (*39*, *40*), we examined the expression of the receptors for these chemoattractants on the ILC progenitor subsets. All of the ILC progenitor populations expressed the CXCR4 protein at similarly high levels (Fig. 4A). More than half of the BM ILCPs, but few CLPs, expressed S1PR1. Some ILC1P, ILC2P, CHILP, and αLPs also expressed S1PR1, but it was ILCPs that expressed S1PR1 at the highest level (Fig. 4B).

The leptin receptor–positive (LEPR^+^) stromal cells not only express *Cxcl12* and *Cxcl14* but also express the S1P synthase *Sphk1* (fig. S10A) (*41*). Therefore, it is expected that CXCR4 ligand and S1P gradients would be formed around the sinusoid niche. In addition, the Itg-α4β1–binding adhesion molecule *Vcam1* is expressed by both LEPR^+^ perivascular stromal cells and sinusoid endothelial cells (fig. S10A), and this would provide adhesion molecules for the α4β1-expressing ILCPs. We created an in vitro BM and blood chemotactic environment using a two-chamber migration system by adding the fluid from mouse BM in top chambers and adding diluted blood plasma to bottom chambers. The BM fluid suppressed the migration of ILCPs, ILC2P, and ILC1P away from top chambers in general, whereas the blood plasma in bottom chambers induced the emigration of ILCPs from top chambers (Fig. 4C). The CXCR4 inhibitor AMD3100 (CXCR4i) mobilized ILCPs from top chambers. This suggests that bloodborne chemoattractants attract ILCPs out of the BM, while CXCR4 ligands retain them in the BM. We further performed the chemotaxis assay for ILC progenitors with S1P in a positive gradient (bottom chambers) and CXCL12 in a negative gradient (top chambers) (Fig. 4D). The most responsive cell population in migration to S1P under this condition was the ILCP, followed by ILC1P and ILC2P, population. The chemotaxis of ILCPs was completely blocked by the S1P receptor inhibitor FTY720. Among the five S1P receptors, ILCPs expressed mainly S1PR1 but hardly S1PR2-5 (fig. S10B). In line with this, a S1PR1-specific inhibitor was effective in blocking the chemotaxis of ILCPs and ILC2P to S1P (Fig. 4E).

**Fig. 5.**
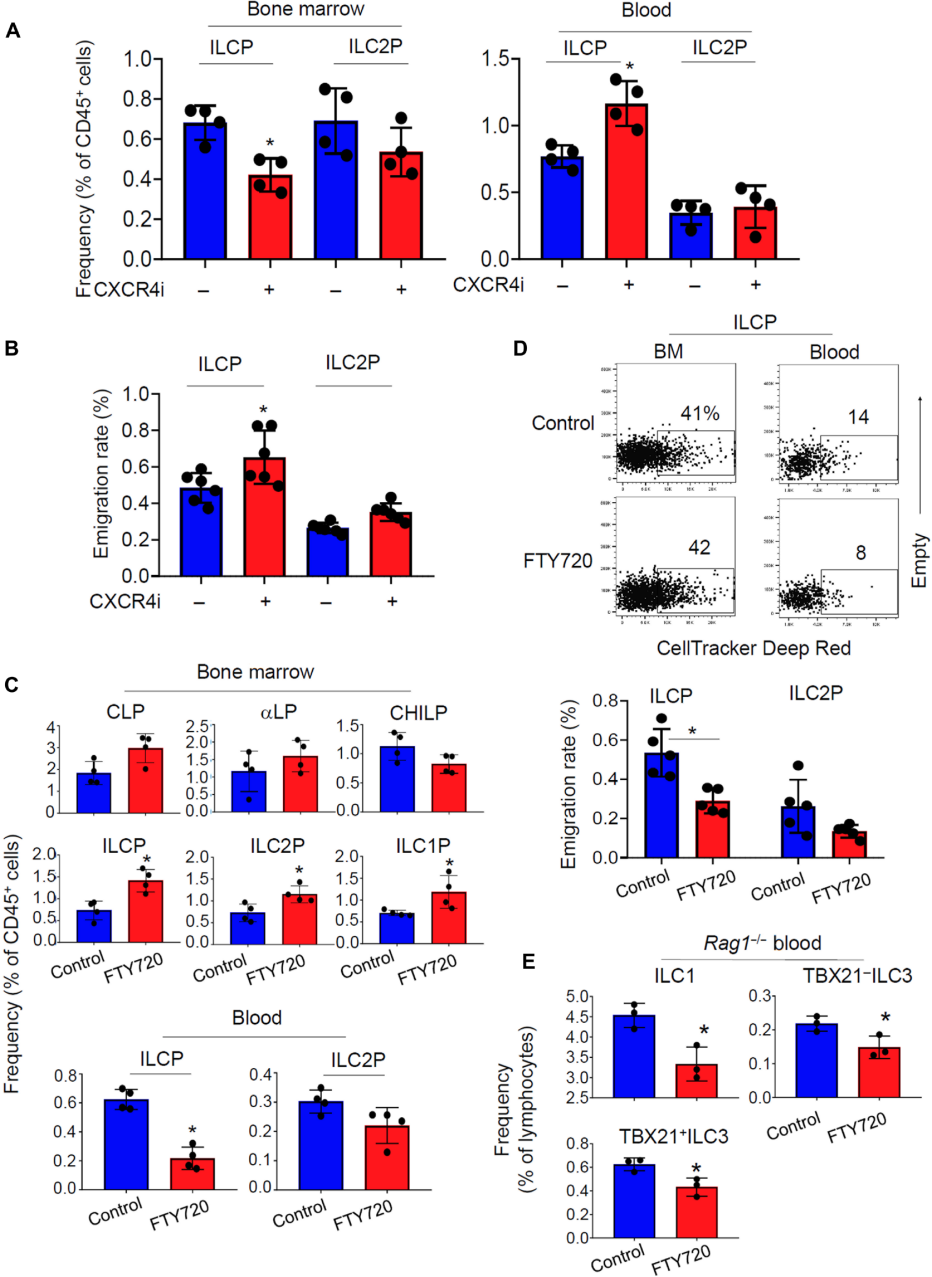
Distinct functions of CXCR4 and S1P receptors in the retention versus emigration of BM ILC progenitors. (**A**) Effect of CXCR4i on the frequency of ILC progenitors in the BM and peripheral blood of *Rag1*^−/−^ mice. (**B**) Effect of CXCR4i on the emigration of BM ILC progenitors in *Rag1*^−/−^ mice. The cells were examined 24 hours after BM CellTracker microinjection. (**C**) Effect of FTY720 on the frequency of ILC progenitors in the BM and peripheral blood of *Rag1*^−/−^ mice. (**D**) Effect of FTY720 on the emigration of BM ILC progenitors in *Rag1*^−/−^ mice. Emigration rates were determined by dividing the frequency of labeled blood cells with that of labeled BM cells in CellTracker-injected mice euthanized 24 hours later. (**E**) Frequency of mature ILCs in the peripheral blood of *Rag1*^−/−^ mice treated with or without FTY720. Pooled data obtained from at least three different experiments (*n* = 4 to 6) are shown. *P < 0.05.

We next performed the pharmacological inhibition of the CXCR4 and S1P receptor functions in vivo (Fig. 5, A to D). The CXCR4i treatment for 12 days decreased the frequency of ILCPs in the BM but increased their frequency in the blood (Fig. 5A and fig. S11A). To determine whether the inhibitors decrease ILCP emigration, we performed the BM CellTracker microinjection and found that the CXCR4i increased the emigration of BM ILCPs (Fig. 5B).

In contrast, the S1PR inhibitor FTY720 increased the frequency of ILCPs in the BM but sharply decreased it in the blood (Fig. 5C and fig. S11B). We also performed the emigration assay and found that FTY720 effectively suppressed the emigration of BM ILCPs (Fig. 5D). FTY720 treatment decreased the numbers of circulating ILC1 and ILC3 (Fig. 5E). We further compared the effects of different S1P inhibitors and found that the S1PR1-specific inhibitor was effective in blocking the emigration of BM ILCPs (fig. S11D). These results emphasize that S1PR1 plays a positive role in the homeostatic emigration of BM ILCPs.

### IL-18, a product of peripheral inflammation, mobilizes BM ILCPs

**Fig. 6.**
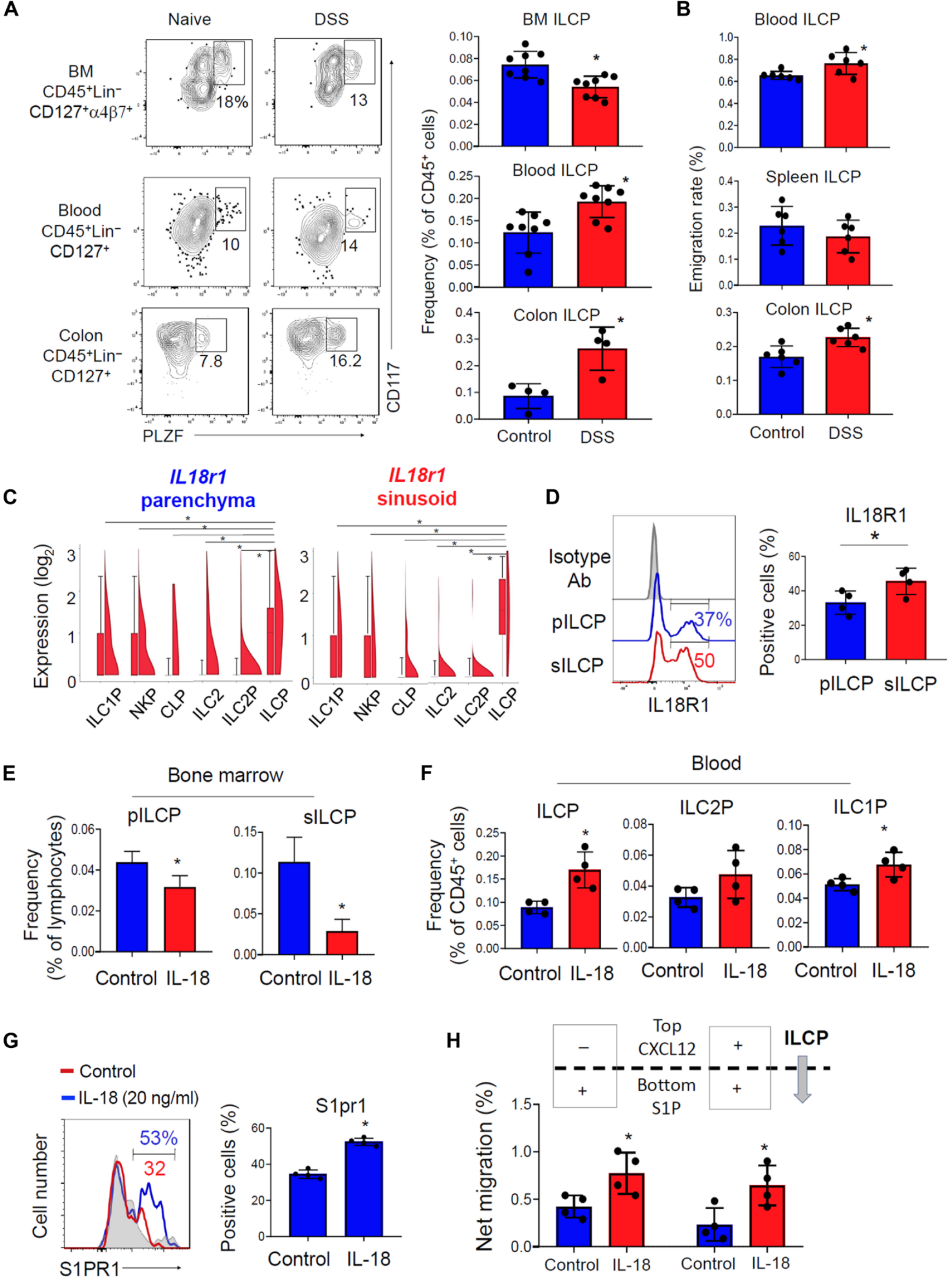
Peripheral inflammation increases IL-18 expression and ILCP emigration. (**A**) Frequency of ILCPs in the BM, blood, and colon of control and DSS-treated mice. (**B**) Emigration rate of BM ILCPs in control and DSS-treated mice. The distribution of CellTracker^+^ cells was examined 48 hours after the labeling in control and DSS-treated mice. (**C**) Expression of *IL18r1* on ILCPs in the BM parenchyma and sinusoids. Violin plots of scRNA-seq data are shown. (**D**) Expression of IL-18R1 on pILCPs versus sILCPs. Ab, antibody. (**E**) Effects of IL-18 treatment on the frequency of pILCPs and sILCPs in the BM. (**F**) Effects of IL-18 treatment on the frequency of ILC progenitors in the peripheral blood. Mice were treated (intraperitoneally) with IL-18 for 4 days and then euthanized 48 hours after the last injection (E and F). (**G**) Effect of IL-18 on the expression of S1PR1. (**H**) Effect of IL-18 on the chemotaxis of ILCP to S1P (+) and CXCL12 (−) gradients. The ILCPs were treated in vitro with IL-18 in the presence of cytokines (stem cell factor and IL-7 at 20 ng/ml) on OP9-DL1 cells as the feeder layer (G and H). Pooled data obtained from at least three different experiments (*n* = 4 to 6) are shown. *P < 0.05.

The composition of the peripheral ILC system and numbers of circulating ILC progenitors dynamically change under inflammatory conditions (*42*–*44*). To determine the impact of peripheral inflammation on the mobilization of BM ILCPs, we induced an acute colitis response in mice with dextran sulfate sodium (DSS) and studied the emigration of BM ILCPs. We found that the frequency of ILCPs was decreased in the BM but increased in the blood and colon tissues by the DSS treatment (Fig. 6A). With the BM CellTracker microinjection approach, we found that the emigration of BM ILCPs to the blood and colon was significantly increased in the DSS-treated mice (Fig. 6B). In contrast, the frequencies of ILCPs in the BM and blood were increased and decreased, respectively, by acute intranasal challenges with *Alternaria alternata* (AA) extract (fig. S12), suggesting that the two inflammatory responses induced by DSS or AA mobilize different types of BM ILC progenitors. We found that sILCPs in both the DSS-treated and untreated mice can make ILC1, ILC2, and CCR6^−^ ILC3 but not CCR6^+^ LTi cells (fig. S13).

One of the cytokine receptors highly expressed by ILCPs is IL-18R1 (Fig. 6C), and this has been observed by others as well (*20*, *27*). Compared to pILCPs, sILCPs expressed *IL18r1* mRNA and protein at higher levels (Fig. 6, C and D). Moreover, a positive correlation between IL-18R1 and S1PR1 on sILCPs, but not pILCPs, was observed (fig. S14A). In contrast, a negative correlation between IL-18R1 and Itg-α4 expression on pILCPs was observed (fig. S14B). Both IL-18R1^+^ and IL-18R1^−^ BM ILCPs can differentiate to ILC1, ILC2, and ILC3 with the IL-18R1^+^ ILCPs being modestly more efficient in making ILC2 (fig. S14C). As reported by others (*45*, *46*), the expression of its ligand *IL18* was increased in the colon of both DSS-treated mice and patients with inflammatory bowel disease (fig. S15, A and B). The functions of IL-18 and its receptor have been unclear in ILCP biology.

Because IL-18R1 is highly expressed by sILCPs and its ligand IL-18 is produced in inflamed intestine, we investigated the possibility that IL-18 may regulate the movement of BM ILCPs in inflammatory conditions. IL-18 administration (intraperitoneally) decreased the numbers of ILCPs in the BM (Fig. 6E) but increased the numbers of ILCPs and ILC1P/ILC1 in the blood (Fig. 6F). The number of BM sILCPs was particularly decreased by the IL-18 administration (Fig. 6E). As a mechanism, we found that IL-18 increased the chemotactic response to S1P and the level of S1PR1 expression on cultured ILCPs (Fig. 6, G and H). Mitogen-activated protein kinase (MAPK) is important for IL-18–induced cell signaling (*47*), and treatment with an MAPK inhibitor abolished the IL-18–induced expression of S1PR1 on ILCPs (fig. S15C). Overall, these results indicate that IL-18 is an inflammatory mobilizer of BM ILCPs.

### sILCPs counteract colitis-associated loss of peripheral ILCs and tissue integrity

**Fig. 7.**
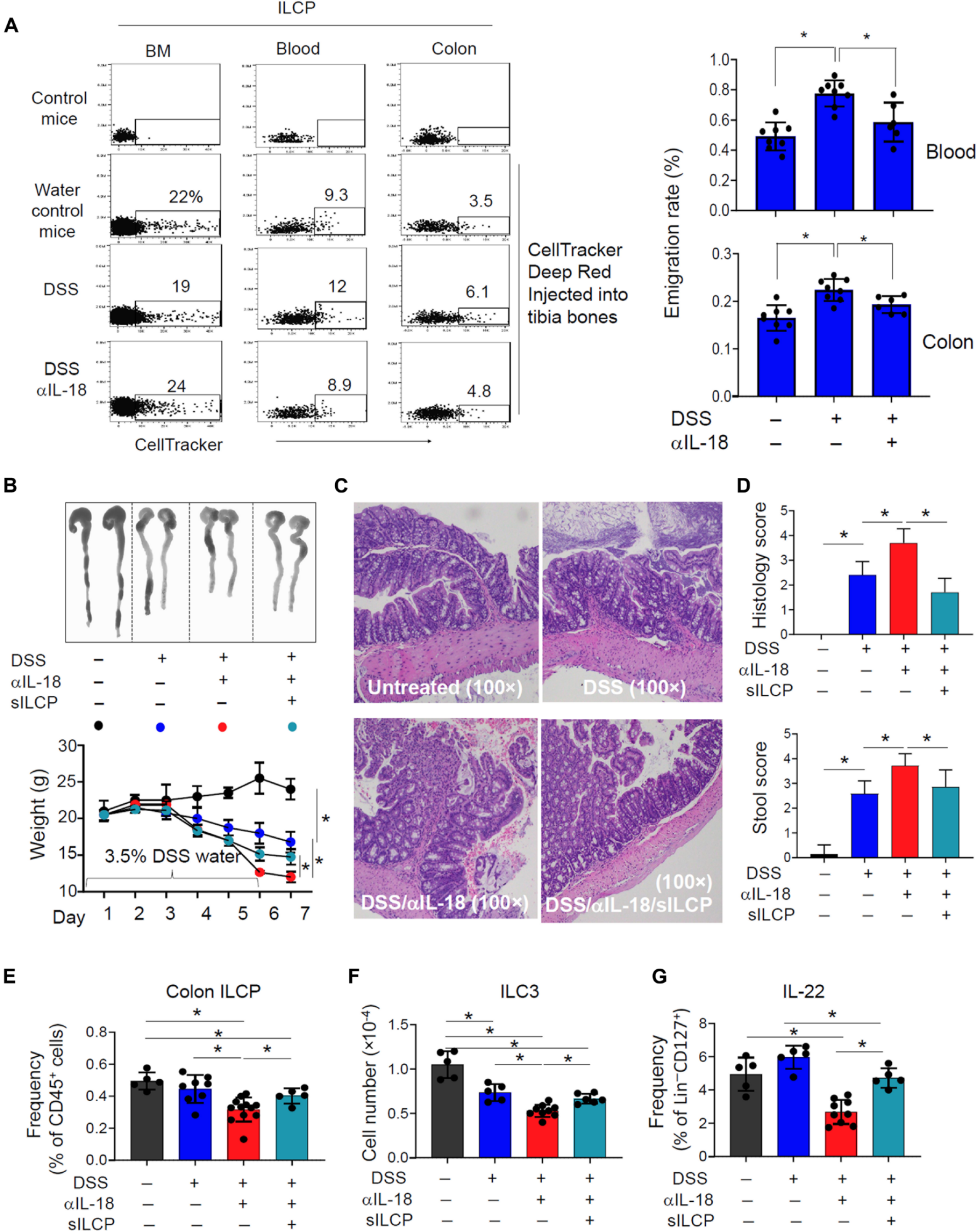
IL-18 mobilizes BM ILCPs and infused BM sILCPs effectively suppresses barrier breach-associated tissue inflammation. (**A**) Effect of IL-18 neutralization on the emigration of ILCPs in the blood and colon of wild-type (WT) C57BL/6 mice treated with DSS. BM CellTracker microinjection was performed on tibia BM cells, and mice were euthanized 48 hours later. (**B** to **G**) Effect of sILCPs on the intestinal inflammation and ILC activity under the suppressed ILCP mobilization condition in DSS-treated *Rag1*^−/−^ mice. Shown are gross morphology and body weight change (B), histological changes (C), histological and stool scores (D), numbers of colon ILCPs (E), numbers of colon ILC3 (F), and IL-22 production by colon ILCs (G). Pooled data obtained from at least three different experiments (*n* = 4 to 8) are shown. *P < 0.05.

We further studied the impact of IL-18 in mobilizing BM ILCPs and determined the function of BM-emigrating ILCPs (i.e., sILCPs) in regulating peripheral inflammation. IL-18 neutralization in the DSS-treated mice decreased the number of emigrated ILCPs in the blood and, to a lesser degree, in the colon (Fig. 7A). This blocking also preferentially increased the relative frequency of the P but decreased that of the S subset of BM ILCPs (fig. S16A), suggesting that IL-18 affects the maturation of trafficking receptors on BM ILCPs. The IL-18 blocking decreased the number of S subset of ILCPs in the blood (fig. S16B), suggesting that IL-18 preferentially induces the mobilization of this subset of BM ILCPs. The IL-18 blocking exacerbated inflammation as evidenced by weight loss, diarrhea, immune cell infiltration, mucosa erosion, and bleeding in *Rag1*^−/−^ mice (Fig. 7, B to D). Thus, the treatment regimen provides a good animal model for ILC insufficiency–associated tissue inflammation. To determine the impact of emigrating BM ILCPs on intestinal inflammation, we infused BM sILCPs intravenously into *Rag1*^−/−^ mice and treated them with DSS (Fig. 7, B to G, and fig. S17A). The infused sILCPs were effective in suppressing tissue injury and various colitis-associated clinical scores (Fig. 7, B to D, and fig. S17B). sILCPs restored the numbers of ILCPs, ILC3, and IL-22–producing ILCs in the colon (Fig. 7, E to G, and fig. S17, C to E). No significant effect of the sILCP infusion was observed on the production of other cytokines such as IL-5, IL-13, and IL-17 by ILCs in this model (figs. S17, D and E, and S18). Infused sILCPs increased CCR6^−^IL-22^+^ cells but not CCR6^+^IL-22^+^ T cells (fig. S19). Overall, these results show that the multipotential sILCPs, mobilized by the inflammasome product IL-18, can prevent or restore inflammation-associated tissue damage.

## DISCUSSION

**Fig. 8.**
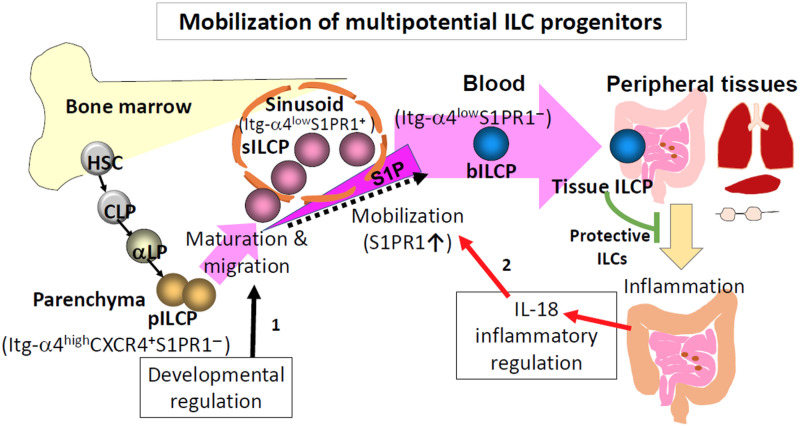
A proposed model for the niche and mobilization of BM ILCPs. pILCPs are developed from early lymphoid progenitors and located in the parenchyma of the BM. Many of these cells are active in cell cycling. pILCPs express CXCR4 and Itg-α4β1, which confer them the microenvironmental tropism within the BM parenchyma. pILCPs developmentally down-regulate Itg-α4β1 to weaken the interaction with parenchymal niche cells and up-regulate S1PR1 to become sILCPs, allowing them to migrate from the parenchyma to the sinusoid niche for emigration. Most sILCPs are in the resting phase of cell cycling and highly expressing IL-18R1 and ICOS. In inflammatory conditions, peripheral signals, such as IL-18, would stimulate IL-18R1^+^ BM ILCPs or sILCPs. This signal further induces the expression of S1PR1 to increase the chemotactic sensitivity of ILCPs to the bloodborne chemoattractant for emigration. Therefore, mobilization of BM ILCPs is controlled in the steady state and further regulated in inflammatory conditions. Mobilized ILCPs in the blood circulation have the Itg-α4β1^low^ S1PR1^−^ phenotype, and these ILCPs can migrate to various peripheral tissues to generate mature ILC1, ILC2, and non-LTi ILC3. The ILCPs, mobilized in inflammatory conditions, have the potential to regulate inflammatory responses and ameliorate tissue damages.

We identified BM-emigrating ILC progenitors with the focus on multipotential ILC progenitors. Two subsets of ILCPs (pILCPs and sILCPs) situated in distinct niches within the BM have been identified (Fig. 8). sILCPs are localized to the sinusoid niche, and pILCPs reside in the broader parenchyma niche. It is the sILCP subset that emigrates the BM to circulate the blood and eventually populate peripheral tissues. The pILCPs and sILCPs differ in the expression of molecules important not only for ILC progenitor differentiation but also for BM retention versus emigration. In inflammation, the mobilization of sILCPs is induced by IL-18, and sILCPs have the capacity to counteract ILC insufficiency–associated inflammatory responses in the gut.

Our study revealed that ILC progenitors are differentially distributed in the BM depending on their stage of maturation. Roughly, two-thirds of BM ILCPs is in the sinusoid area and the other one-third is in the parenchyma away from the sinusoid vasculature. sILCPs are in the BM sinusoid niche, and this appears to be important for them to be mobilized or become ready for emigration in response to external cues. In contrast, pILCPs are in the nonsinusoid areas of the central niche. pILCPs and sILCPs are highly related to each other in their overall transcriptome profile but are different in the expression of certain genes that are important for their tissue localization and trafficking.

Most pILCPs were Itg-α4^hi^S1PR1^−^ cells, whereas sILCPs included many Itg-α4^low^S1PR1^+^ cells and some transitional Itg-α4^hi^S1PR1^+^ cells. In the peripheral blood, the circulating ILCPs were predominantly Itg-α4^low^S1PR1^−^ cells with small numbers of Itg-α4^hi^S1PR1^−^ and Itg-α4^low^S1PR1^+^ cells. It appears that sILCPs emigrate the BM mainly as Itg-α4^low^S1PR1^+^ cells and then lose the surface S1PR1 expression in the blood to become circulating Itg-α4^low^S1PR1^−^ cells. Because all ILCPs express Itg-β1, which pairs with Itg-α4, most Itg-α4^hi^ ILCPs cells should express α4β1. Itg-α4β1 would work with CXCR4 to position ILCPs in the appropriate niches in the BM. VCAM-1 is expressed on the BM stromal and endothelial cells, and fibronectin is a part of the BM parenchymal ECM (*48*, *49*). Therefore, Itg α4β1^hi^ pILCPs would adhere to BM tissue cells and parenchyma ECM. These trafficking receptors not only regulate the positioning of ILCPs within the BM but also provide useful markers to identify multiple BM ILCP subsets at distinct stages of differentiation and migration.

The results shed light on the mechanism of ILCP retention versus emigration from the BM. The chemoattractants that trigger CXCR4 and S1PRs appear to play major roles in the retention and emigration of BM ILCPs, in a manner somewhat similar to their roles in regulating the retention and egress of hematopoietic stem and progenitor cells (*50*). CXCR4 has two ligands, CXCL12 and CXCL14, which are most highly expressed by the BM LEPR^+^ MSCs in the central niche. The results from our chemotaxis and in vivo CXCR4 and S1PR inhibition studies indicate that the CXCR4 ligands appear to keep the ILCPs within the BM. The S1PR1 expression is increased on sILCPs, and this makes the cells sensitive to the bloodborne chemoattractant S1P. However, the expression of CXCR4 is uniformly high on both pILCPs and sILCPs. While there are multiple receptors for S1P (S1PR1-S1PR5), ILCPs mainly express S1PR1, which induces the emigration of ILCPs to the blood circulation. The fine balance between the CXCR4 and S1PR ligands, along with the expression levels of Itg-α4 versus S1PR1, appears to decide whether a particular ILCP should be retained or pushed out for emigration from the sinusoid niche. CD69, induced upon lymphocyte activation, inhibits the function of S1PR1 (*51*). This makes developing and activated lymphocytes retained in the thymus and lymph nodes. Both pILCPs and sILCPs expressed comparable levels of CD69, and therefore, CD69 expression by itself does not account for the different emigration abilities of pILCPs and sILCPs. Further studies may be performed to rule out the potential function of CD69 in regulating the emigration of BM ILCPs.

We found intrinsic and extrinsic signals that control the emigration of BM ILCPs. As an intrinsic factor, the regulated and cell stage–dependent expression of the BM retention and emigration-associated trafficking receptors (i.e., Itg-α4β1, CXCR4, and S1PR1) can be important. As an external factor in inflammatory conditions, IL-18, which is produced as the product of inflammation, increases the expression of S1PR1 and the emigration of BM ILCPs. While both sILCPs and pILCPs express IL-18R1, it is expected that IL-18 preferentially affects sILCPs over pILCPs because they are located in sinusoids, which are readily accessible by circulating factors in the blood. Moreover, IL-18R1 is expressed by ~60% sILCPs and ~35% pILCPs. Many sILCPs express both IL-18R1 and S1PR1 and therefore would be efficient in sensing the peripheral IL-18 signal and emigrating the BM.

Our results indicate that ILCPs, ILC1P, and ILC2P have distinct emigration behaviors. The emigration rate at steady state was high for BM ILCPs and ILC1P but low for ILC2P. Thus, ILCPs and ILC1P are preferentially mobilized in the steady state. It has been shown that BM ILC2P and ILC2 are mobilized by IL-33 under lung allergy conditions (*44*), which, together with the expansion of preexisting tissue ILC2, is in line with the sharp increase of ILC2 in peripheral tissues during helminth infection or allergic inflammation (*52*, *53*). We observed that DSS-induced gut inflammation increased the emigration of ILCPs but not ILC2P, whereas AA-induced lung type 2 inflammation, which increases blood ILC2P, had a moderate negative effect on the frequency of circulating ILCPs. Potential explanations for the different migration behavior include differential positioning of the ILC progenitors in the BM and heterogeneous expression of S1PR1 by these ILC progenitor subsets. ILC2P express S1PR1 at relatively low levels compared with ILCPs and ILC1P and primarily reside in the parenchyma rather than sinusoid niche. More studies are needed to understand the emigration mechanisms for the single potential ILC progenitors.

The emigration of sILCPs would have important effects on the homeostasis of peripheral ILC populations. This process would be important to slow down both age-dependent and induced depletion of peripheral ILCs following immune and inflammatory responses (*17*, *18*, *25*, *54*). Continuous influx of ILC progenitors would be important to ensure the maintenance of the finite ILC progenitor pool in peripheral tissues. sILCPs, as reported previously for ILCPs in general (*10*), make ILC1, ILC2, and non-LTi ILC3, but not CCR6^+^ LTi cells. In this regard, we found that IL-18 is required for optimal numbers of ILCPs and non-LTi ILC3 that produce IL-22 in inflamed intestine. While the IL-18 function is context dependent, many observed that tissue inflammation is worsened in IL-18 deficiency (*55*, *56*). We used a colitis model in *Rag1*^−/−^ mice under an IL-18 blockade condition, which led to both decreased ILCP mobilization and exacerbated inflammation in the colon. sILCPs, when infused into the blood circulation, were effective in restoring the numbers of ILCPs and IL-22–producing ILC3 in the colon. Moreover, sILCPs were effective in suppressing the inflammation and tissue damage in the colon. Thus, mobilized BM ILCPs have the potential to effectively prevent inflammatory responses and tissue damages by generating protective ILCs.

In sum, our results provide fundamental insights into the mobilization of the multipotential BM ILC progenitors in the steady state and under inflammatory conditions. Potential mechanisms and cell-extrinsic signals that control the mobilization of ILCPs were identified. ILC insufficiency would be a major problem in many pathological conditions such as infection, inflammation, and natural and induced immunodeficiency. We envision that the outcomes from this study provide applicable ideas to control the number of circulating ILCPs and use mobilized ILC progenitors for therapeutic purposes to normalize ILC insufficiency–associated pathological conditions in peripheral tissues.

## MATERIALS AND METHODS

### Study design

This study was performed to understand the control mechanisms for the mobilization of BM ILCPs and to identify molecular features of the mobilized ILCPs under steady state and inflammatory conditions. We performed sinusoid cell labeling, in-bone dye labeling for tracking mobilized BM ILCPs in the periphery, scRNA-seq, immunofluorescence staining of the BM, and parabiosis experiments as described below. Sufficient numbers of samples as indicated in each figure were used for statistical power. Most in vivo experiments were repeated three times unless indicated otherwise. All relevant data were included for calculation, and the study was not blinded or randomized.

### Animals

The animal protocols for the study were approved by the Animal Care and Use Committees at University of Michigan (PRO00009958). C57BL/6 mice were from Harlan, and CD45.1 (The Jackson Laboratory, stock 006584), PLZF-GFP (green fluorescent protein) (The Jackson Laboratory, stock 024529), and *Rag1*^−/−^ (The Jackson Laboratory, stock 002216) mice on the C57BL/6 background were originally from The Jackson Laboratory. *Rag2*^−/−^*IL2rg*^−/−^ mice on the C57BL/6 background were from Taconic Biosciences (4111-F). All mice were kept under a specific pathogen–free condition on a regular rodent chow ad libitum on the 12-hour dark and 12-hour light cycle. The experiments were performed on 6- to 8-week-old male and female mice. When indicated, mice were injected daily with mouse IL-18 (200 ng per mouse, i.p.; BioLegend) for 4 days, and mice were euthanized 48 hours after the last injection.

### ILC progenitor isolation

BM cells were flushed from femurs and tibias with phosphate-buffered saline (PBS; pH 7.3) supplemented with 0.5% bovine serum albumin (BSA) and 0.1 mM EDTA. In some emigration experiments, cells were also flushed from chopped skull bones. Red blood cells were lysed with ammonium chloride potassium (ACK) lysis buffer. The spleen and mesenteric lymph nodes were made into single-cell suspensions by filtration through fine meshes. The skin and gonadal white adipose tissue were minced and digested with collagenase IV for 45 min (1.5 mg/ml; Worthington, Lakewood, NJ). Intestine tissues were cut open longitudinally and washed with cold PBS. Peyer’s patches were removed, and then tissues were cut into 1- to 2-cm-long pieces and treated three times with Hanks’ balanced salt solution containing 1 mM EDTA, 2% Hepes, and NaHCO_3_ (0.35 g/liter) to remove epithelial cells. The remaining tissues were digested by collagenase IV (1.5 mg/ml; Worthington, Lakewood, NJ) containing 10% newborn calf serum for 45 min at 37°C to make lamina propria cell suspensions. Lung and liver tissues were cut into small pieces with scissors and digested with collagenase IV (1.5 mg/ml; Worthington). The digested solution was further homogenized through iron meshes and lysed with ACK buffer. Intestinal and lung cell suspensions were processed to enrich leukocytes with density cut centrifuge on 40/80% Percoll layers for 30 min. BM parenchyma and sinusoid ILC progenitors were sorted after sinusoid labeling. Lin^−^ CD127^+^ CD117^+^ PLZF-GFP (or PD-1)^+^ ILCPs were sorted (~95% pure) from the BM of naïve or DSS-treated mice with a flow cytometry sorter (FACSMelody, BD Biosciences).

### Flow cytometry

Gating information for ILC progenitor subsets and mature ILCs (NK, ILC1, ILC2, and ILC3) has been previously described (*57*). The definitions for flow cytometry of the BM ILC progenitors are described in table S1. Cells were stained with antibodies to surface antigens, such as CD25 (clone 3C7), CD45.2 (104), CD45.1 (A20), CD90.2 (53-2.1), CD127 (A7R34), SCA-1 (D7), KLRG1 (2F1), FLT3 (A2F10), CD117 (ACK2), NKp46 (29A1.4), IL-23R (12B2B64), CD122 (5H4), PD-1 (29F.1A12), CD159a (16A11), Ly6C (HK1.4), CD28 (37.51), S1PR1 (713412), CXCR4 (L276F12), CCR2 (QA18A56), CXCR6 (SA051D1), ICOS (7E.17G9), CCR6 (29-2 L17), CD69 (H1.2F3), and lineage antigens (CD3ε, CD4, CD8, CD11b, CD11c, CD19, B220, Gr-1, NK1.1, and TER119). Expression of S1PR1 (713412), CXCR4 (L276F12), CCR2 (QA18A56), CXCR6 (SA051D1), CCR6 (29-2 L17), Itg-β1 (HMβ1-1), and α4β7 (DATK32) was assessed in a three-step staining with primary rat antibody, biotinylated anti-rat immunoglobulin G (IgG) 2a/b/c (clone MRG2a-83/2b-85/2c-67) or biotin goat anti-hamster IgG (Poly4055), and fluorescent dye–conjugated streptavidin. For ILC1P and NKP cells, anti-NK1.1 was omitted from the lineage cocktail. Cells were fixed and permeabilized with the Transcription Factor Staining Buffer Kit (Tonbo Biosciences) for further staining of T-BET (eBio-4B10), GATA3 (TWAJ), RORγt (AFKJS-9), PLZF (9E12), and EOMES (Dan11mag). For intracellular staining of cytokines, cells were stained for surface markers, followed by activation with phorbol 12-myristate 13-acetate (50 ng/ml; Sigma-Aldrich), ionomycin (1 μg/ml; Sigma-Aldrich), and monensin (2 μM; Sigma-Aldrich) for 3 to 4 hours. Cells were fixed with 1% paraformaldehyde for at least 2 hours, then permeabilized with saponin buffer, and stained for IL-22 (Poly5164), IL-17 (TC11-18H10.1), IL-5 (TRFK5), and IL-13 (W17010B). Most of the antibodies were from BioLegend or eBioscience unless indicated otherwise. Stained cells were acquired on a NovoCyte Flow Cytometer (ACEA Biosciences Inc.). For cell cycle analysis, DRAQ7 (BD Biosciences) was used to quantify the DNA content. Briefly, single-cell suspension was stained for surface markers, fixed with drop-by-drop 70% ice-cold ethanol for 30 min, and then stained with DRAQ7 for flow cytometry analysis.

### ILC progenitor dynamic in parabiosis mice

Upon weaning at 3 weeks of age, CD45.1 wild type (WT) and CD45.2 WT were cohoused for 2 weeks and then surgically paired to make parabiosis mice as described previously (*58*). Parabiosis mice were euthanized 2 months after surgery, and the numbers of ILC progenitors in the BM, blood, and colon were examined by flow cytometry. The parabiosis rate (% donor derived) was calculated on the basis of the frequency of donor- and host-derived ILCs and their progenitors in each organ for each subset.

### BM sinusoid cell labeling and emigration study

For BM sinusoid cell labeling, WT or PLZF-GFP mice were injected intravenously with anti-CD45.2^−^ AmCyan antibody (1 μg; clone 104, BioLegend). Mice were euthanized 2 min after the injection, and BM cells were stained for flow cytometry to determine the numbers of indicated ILC progenitor cells. For BM cell labeling with CellTracker Deep Red (Thermo Fisher Scientific) (*33*), the skin above the skull or tibia was cut open, and small holes were made with a 30-gauge needle. Then, 3 μl of diluted CellTracker was slowly injected into each site by using a 5-μl syringe (#65 Hamilton Co., Reno, NV, USA) and a custom 34-gauge blunt needle (RN 0.375″ PT3, Hamilton). The skin incision was sutured, and mice were euthanized for flow cytometry analysis 24 to 48 hours after the microinjection. Emigration rate (%) for each organ was based on the ratio: [Frequency of CellTracker-labeled cells of CD45^+^ cells in blood or colon]/[Frequency of CellTracker-labeled cells of CD45^+^ cells in the BM].

### Confocal microscopy of ILC progenitors in the BM

Bone preparation and immunostaining were performed as previously described (*59*). Briefly, femurs from PLZF-GFP mice were fixed in 4% paraformaldehyde overnight with gentle rocking at 4°C. After fixation, bones were frozen in optimal cutting temperature compound (Sakura). Frozen tissue blocks were cut into 10-μm sections, permeabilized with PBS containing 10% dimethyl sulfoxide and 0.5% NP-40 (staining buffer) overnight at room temperature. Then, tissue sections were stained with anti-laminin (clone AL-4, rat IgG, R&D Systems) in the same medium for 4 hours at room temperature and washed. The sections were further stained with a secondary antibody (goat anti-rat IgG) for 1 hour at room temperature. After washing and blocking with 10% rat serum in the staining buffer, tissue sections were stained with Hoechst 33342 and secondary antibodies to the lineage cocktail (CD3, CD19, CD11c, and CD11b) and GFP. The images were obtained with a Nikon A1 confocal system equipped with gallium arsenide phosphide detectors for high sensitivity.

### Chemotactic behavior of BM ILC progenitors

To obtain the BM chemoattractant fluid, two femurs and two tibia bones were flushed in a total of 1-ml chemotaxis medium (RPMI 1640 and 0.5% BSA). Blood plasma was used as 1:10 diluted fluid in the chemotaxis medium. Freshly isolated or cultured Lin^−^CD127^+^ BM cells (5 × 10^5^ per well) were resuspended in the chemotaxis medium and placed in the upper chamber of 24-well Transwells (5.0-μm pore size; Corning, Corning, NY) along with or without murine CXCL12 (100 ng/ml; BioLegend) in the same chambers to form a negative gradient of CXCL12. The cells were allowed to migrate to the lower chamber containing S1P (100 nM; Cayman Chemical) for 3 hours at 37°C in 5% CO_2_ atmosphere. The cells in the lower chamber were collected; stained with antibodies to identify ILCP, ILC1P, ILC2P, and other ILC progenitor subsets; and counted by a time-based flow cytometry (*21*).

### scRNA-seq data analysis

Sorted BM sinusoid and parenchyma Lin^−^CD127^+^ cells were stained with the Mouse Immune Single-Cell Multiplexing Kit and loaded onto a BD Rhapsody cartridge. cDNA synthesis was performed using the BD Rhapsody Express Single-Cell Analysis System, and gene expression libraries were prepared using the Whole Transcriptome Analysis Amplification Kit (all from BD Biosciences). The raw FASTQ data were aligned to mouse grcm38 genome and processed by the Seven Bridges Platform (www.sevenbridges.com/) for sample demultiplexing and the generation of expression matrix files. The scRNA-seq data were further analyzed with SeqGeq genomic tool version 9.0 (FlowJo, LLC). Multistep filtering (https://docs.flowjo.com/seqgeq/quality-control/) in SeqGeq was performed to remove low-quality cells (dead cells, debris, and low RNA-expressing cells) before performing dimensionality reduction on *Il7r*-expressing cells to generate an unbiased t-SNE plot. A total of 22,156 unique molecular identifiers (or genes) were detected. A total of 1643 cells were initially identified on the basis of unique barcodes, and 1210 cells passed the filtering. Last, 493 cells from parenchyma and 717 cells from sinusoids were analyzed for cell frequency and transcriptome. A principal components analysis (PCA) reduction (15 dimensions) was performed followed by a t-SNE dimensionality reduction. *k*-means filtering (*k* = 67) was performed to cluster the cells into six populations based on the PCA variability and PhenoGraph algorithm (*60*). The six clusters identified by PhenoGraph were overlayed onto the t-SNE map. The Cluster Explorer plugin was used to characterize the immunophenotype of each cluster. Mean cluster transcript expression plots and expression heatmaps of differentially expressed genes were prepared with the SegGeq and Prism S/W. The Monocle plugin was used to perform the single-cell trajectory analysis and define transitional states of differentiation processes. Gene Ontology (molecular function) and KEGG (Kyoto Encyclopedia of Genes and Genomes) pathway analyses were performed using DAVID (Database for Annotation, Visualization, and Integrated Discovery, version 6.8; https://david.ncifcrf.gov/tools.jsp) based on differentially expressed genes between pILCPs and sILCPs. The data are deposited in National Center for Biotechnology Information (NCBI) Gene Expression Omnibus (GSE193835).

### In vitro and in vivo ILC progenitor differentiation

Sorted parenchyma and sinusoid Lin^−^ CD127^+^ CD117^+^ PD-1^+^ IL-18R1^+/−^ ILCP cells were cocultured with OP9-DL1 cells in complete Dulbecco’s minimum essential medium in the presence of murine IL-7 and stem cell factor (20 ng/ml each). The OP9-DL1 cells were pretreated with mitomycin C (50 ng/ml) for 25 min to stop cell division before the culture. Half of the culture medium was replaced every 3 days, and cultured cells were harvested on days 10 to 12. When indicated, recombinant IL-18 (BioLegend) and U0126 (250 nM; AS1517499, Cayman Chemical) were added to the culture. For in vivo differentiation, parenchyma and sinusoid Lin^−^CD127^+^CD117^+^PD-1^+^ ILCP cells, sorted from the BM of CD45.1^+^ and CD45.2^+^ congenic mice, respectively, were coinjected at 1:1 ratio (4 to 5000 per mouse) intravenously into *Rag2*^−/−^*IL2rg*^−/−^ mice. The cotransferred mice were euthanized 4 weeks later, and the frequency and numbers of ILC1/2/3 subsets and ILC progenitors in various organs and blood were determined.

### Acute DSS-induced intestinal inflammation and AA extract–induced lung inflammation

For DSS-induced colitis, WT or *Rag1*^−/−^ mice were treated with 3.5% DSS in drinking water for 6 days and then with regular water for another day before termination. Mice were monitored for weight change and clinical activity, which is defined by the following criteria: normal (score of 0), soft stool in a normal shape (1), loose stool with high water content (2), diarrhea (3), and rectal bleeding (4). Sinusoid antibody-labeled Lin^−^ CD127^+^ CD117^+^ PLZF (GFP)^+^ sILCPs were sorted from mice injected intravenously with anti-CD45.2 and euthanized 2 min later. CD45.2^+^ sILCPs (4 to 5000 per mouse) were transferred intravenously into *Rag1*^−/−^ mice on day 2. Mice were injected intraperitoneally with a neutralizing anti–IL-18 (YIGIF74-1G7, BioXcell) or isotype control on days 1 and 4 during the experiment, and mice were euthanized on day 7. *Rag1*^−/−^ mice were challenged intranasally with AA extract (50 μg in 50 μl of PBS) on days 1 and 3. Mice were euthanized on day 6, and blood and BM cells were harvested for flow cytometry analysis of ILC progenitors.

### Statistical analysis

A power calculation was done to determine the group size for in vivo experiments to detect significant differences between two and four groups with 60 to 80% power, an effect size of 0.5, and a two-sided 0.05 significance level. Statistical significance was calculated by unpaired two-tailed *t* test with Prism (version 8.0, GraphPad Software) to compare two experimental groups or one-way analysis of variance (ANOVA) for multiple comparisons. *P* values < or = 0.05 were considered significant.

## Notes

View/request a protocol for this paper from *Bio-protocol*.
