# Supplementary material for: Identification of the niche and mobilization mechanism for tissue-protective multipotential bone marrow ILC progenitors
Source: Sci Adv. 2022 Nov 23;8(47):eabq1551. doi: 10.1126/sciadv.abq1551 (PMC9683709; doi:10.1126/sciadv.abq1551)
Supplement: Supplementary file 1 — Table S1 Figs. S1 to S19 [file sciadv.abq1551_sm.pdf]

Science Advances
AAAS

Supplementary Materials for

Identification of the niche and mobilization mechanism for tissue-protective multipotential bone marrow ILC progenitors

Qingyang Liu *et al.*

Corresponding author: Chang H. Kim, chhkim@umich.edu

Sci. Adv. **8**, eabq1551 (2022)
DOI: 10.1126/sciadv.abq1551

This PDF file includes:

Table S1
Figs. S1 to S19

Supplementary Materials

Supplementary Table 1: Surface markers of ILC subsets and gating strategy for flow cytometry

Subsets	Gating strategy
Mature ILC1	$CD45^+$ Lin^- $CD127^+$ $CD90^+$ $ROR\gamma t^-$ $GATA3^-$ $Tbet^+$
Mature ILC2	$CD45^+$ Lin^- $CD127^+$ $CD90^+$ $ROR\gamma t^-$ $Tbet^-$ $GATA3^+$
Mature ILC3	$CD45^+$ Lin^- $CD127^+$ $CD90^+$ $ROR\gamma t^+$ $Tbet^{+/-}$
Non-LTi ILC3	$CD45^+$ Lin^- $CD127^+$ $CD90^+$ $ROR\gamma t^+$ $Tbet^-$ $CCR6^-$
LTi ILC3	$CD45^+$ Lin^- $CD127^+$ $CD90^+$ $ROR\gamma t^+$ $Tbet^-$ $CCR6^+$
BM CLP	$CD45^+$ Lin^- $CD127^+$ $SCA\text{-}1^{int/+}$ $Flt3^+$ $\alpha 4\beta 7^-$
BM αLP	$CD45^+$ Lin^- $CD127^+$ $SCA\text{-}1^{int/+}$ $Flt3^-$ $\alpha 4\beta 7^+$
BM CHILP	$CD45^+$ Lin^- $CD127^+$ $SCA\text{-}1^{int/+}$ $Flt3^-$ $\alpha 4\beta 7^+$ $CD25^-$ $CD117^+$
BM ILCP	$CD45^+$ Lin^- $CD127^+$ $CD117^+$ PLZF (or $PD\text{-}1^+$) $\alpha 4\beta 7^+$
BM ILC2P	$CD45^+$ Lin^- $CD127^+$ $SCA\text{-}1^{int/+}$ $Flt3^-$ $\alpha 4\beta 7^+$ $CD25^+$ $CD117^-$
BM ILC1P*	$CD45^+$ Lin^- $CD127^+$ CD49a (or $CD122^+$) $EOMES^-$ $NK1.1^+$ $NKp46^+$
BM NKP*	$CD45^+$ Lin^- $CD122^+$ $NK1.1^-$ $DX5^-$

The lineage cocktail (Lin) includes antibodies to CD3ε, CD4, CD8, TCRγδ, CD11b, CD11c, CD19, B220, Gr-1, NK1.1, and Ter119. For ILC1P and NKP cells, anti-NK1.1 was omitted.
*This combination does not distinguish ILC1P and mature ILC1 cells in peripheral tissues.

Supplementary Figures

A

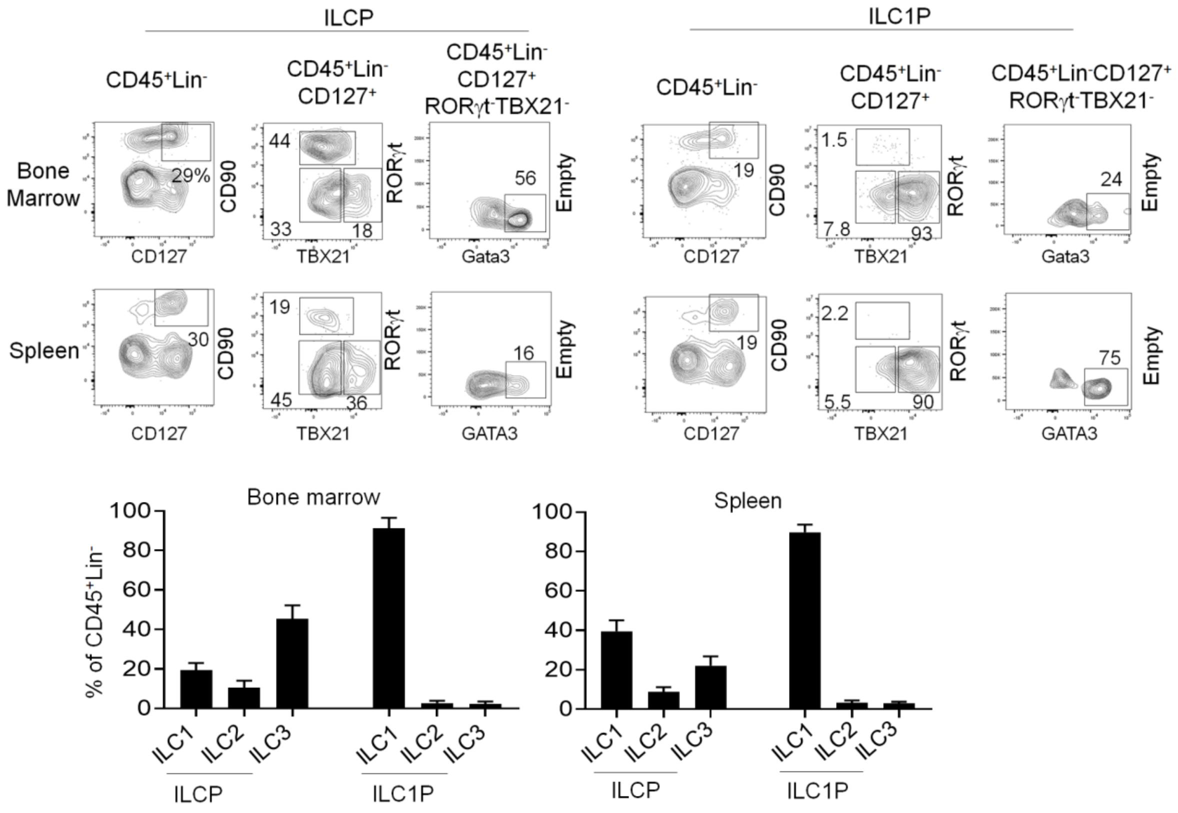

B

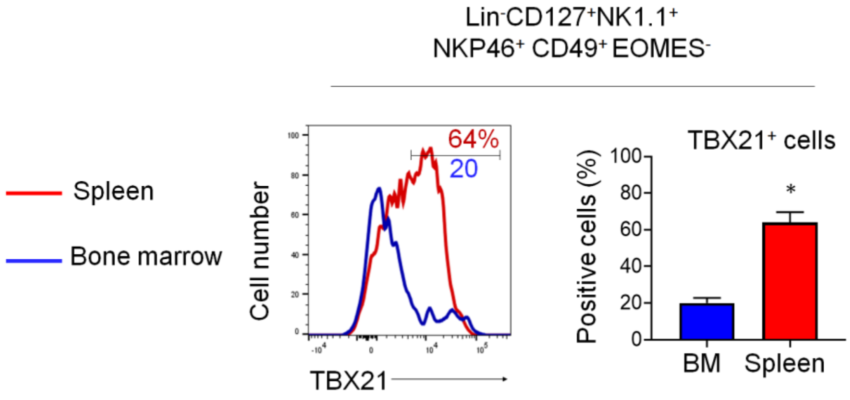

Supplementary Figure S1. Validations ILCP and ILC1P progenitor populations. (A) Comparison of Lin$^-$CD127$^+$CD117$^+$PLZF$^+$ ILCPs and Lin$^-$CD127$^+$CD49a$^+$NK1.1$^+$NKp46$^+$ ILC1P in the BM and spleen for their differentiation potential. The populations were cultured on OP9-DL1 cells in the presence of IL-7, SCF for mature ILCs production. (B) TBX21 phenotype of Lin$^-$ CD127$^+$ NK1.1$^+$ NKP46$^+$ CD49$^+$ EOMES$^-$ cells from the BM and spleen. Pooled data obtained from four to five different pairs are shown. *Significant differences ($P < 0.05$).

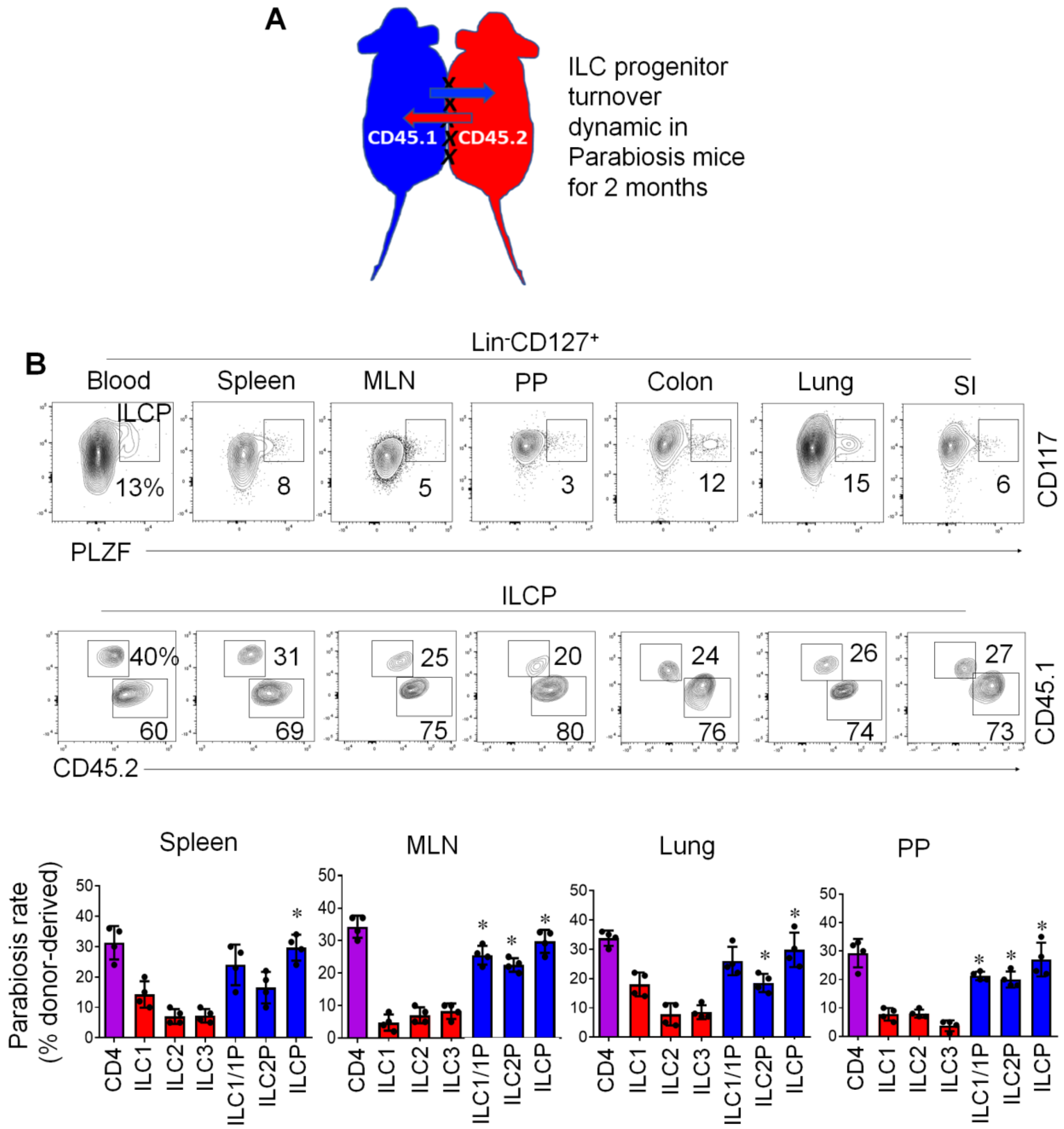

Supplementary Figure S2. The parabiosis rates of mature ILCs and ILC progenitors. (A) Parabiosis between CD45.2 WT and congenic CD45.1 WT mice was maintained for 2 months and examined for relative population of donor versus host mature ILCs and ILC progenitor subsets in various tissues. (B) Representative plots for frequency of ILCPs in indicated tissues of CD45.2 mice in parabiosis with CD45.1 mice and graphs for frequency of indicated donor-derived ILC subsets in different tissues are shown. Pooled data obtained from four to five different pairs are shown. *Significant differences ($P < 0.05$) from WT. Abbreviations for tissues: mesenteric lymph nodes (MLN) and Peyer's patches (PP).

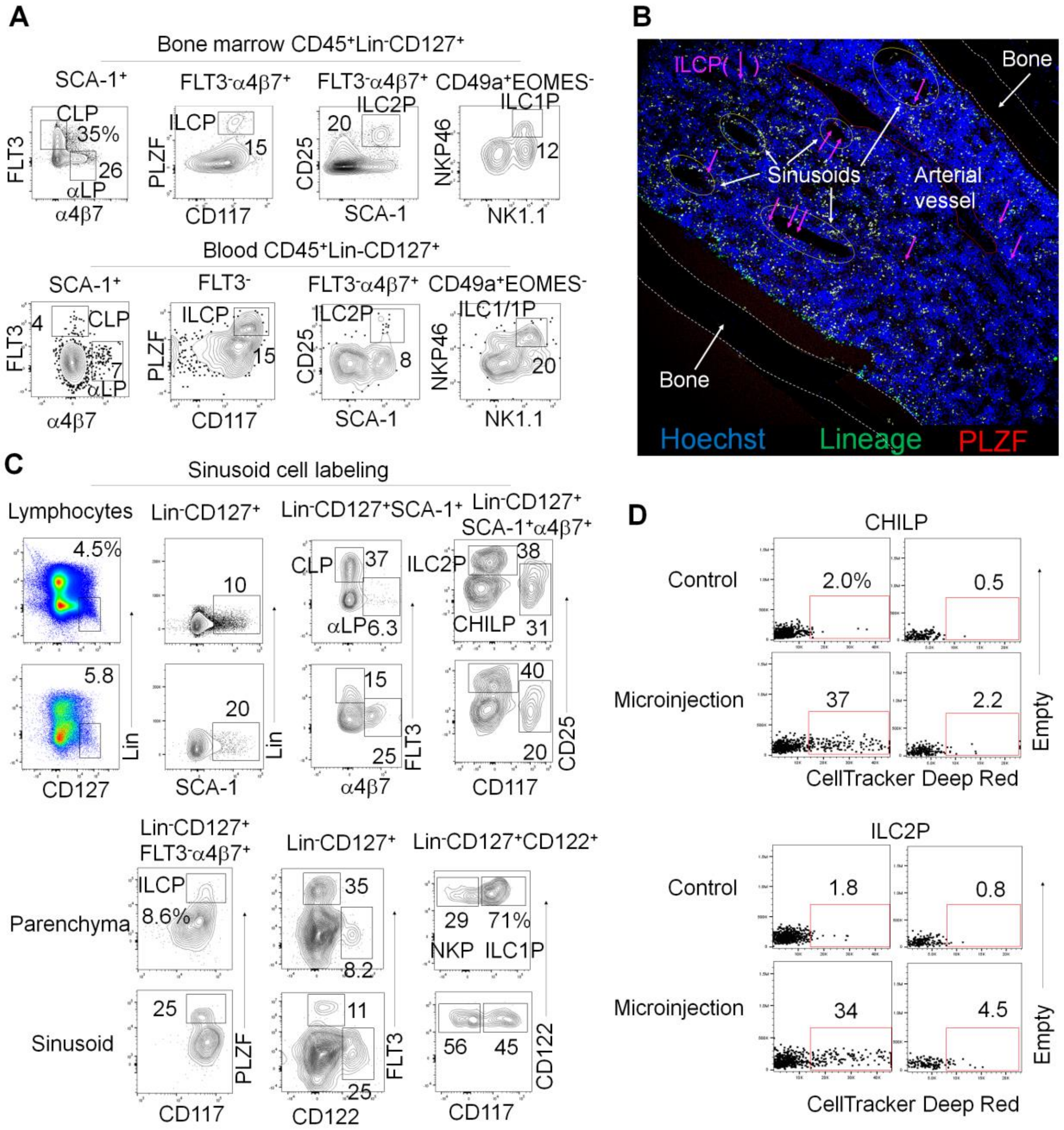

Supplementary Figure S3. The niche, blood circulation and emigration of ILC progenitors. (A) Representative flow data for ILC progenitor subsets in the BM and peripheral blood. (B) Immunofluorescent staining of a frozen bone marrow section. The sections were stained with Hoechst, lineage cocktails Abs, and anti-GFP for PLZF expression to visualize Lin$^-$PLZF$^+$ ILC progenitors. (C) Representative flow data for the BM ILC progenitors in the parenchymal and sinusoid niches. A rapid (2 min) sinusoid labeling with anti-CD45 was performed. (D) Representative flow data for dye (CellTracker Deep Red) labeled BM ILC progenitors in the peripheral blood in control and dye-injected mice. Representative data from at least 3 independent experiments are shown.

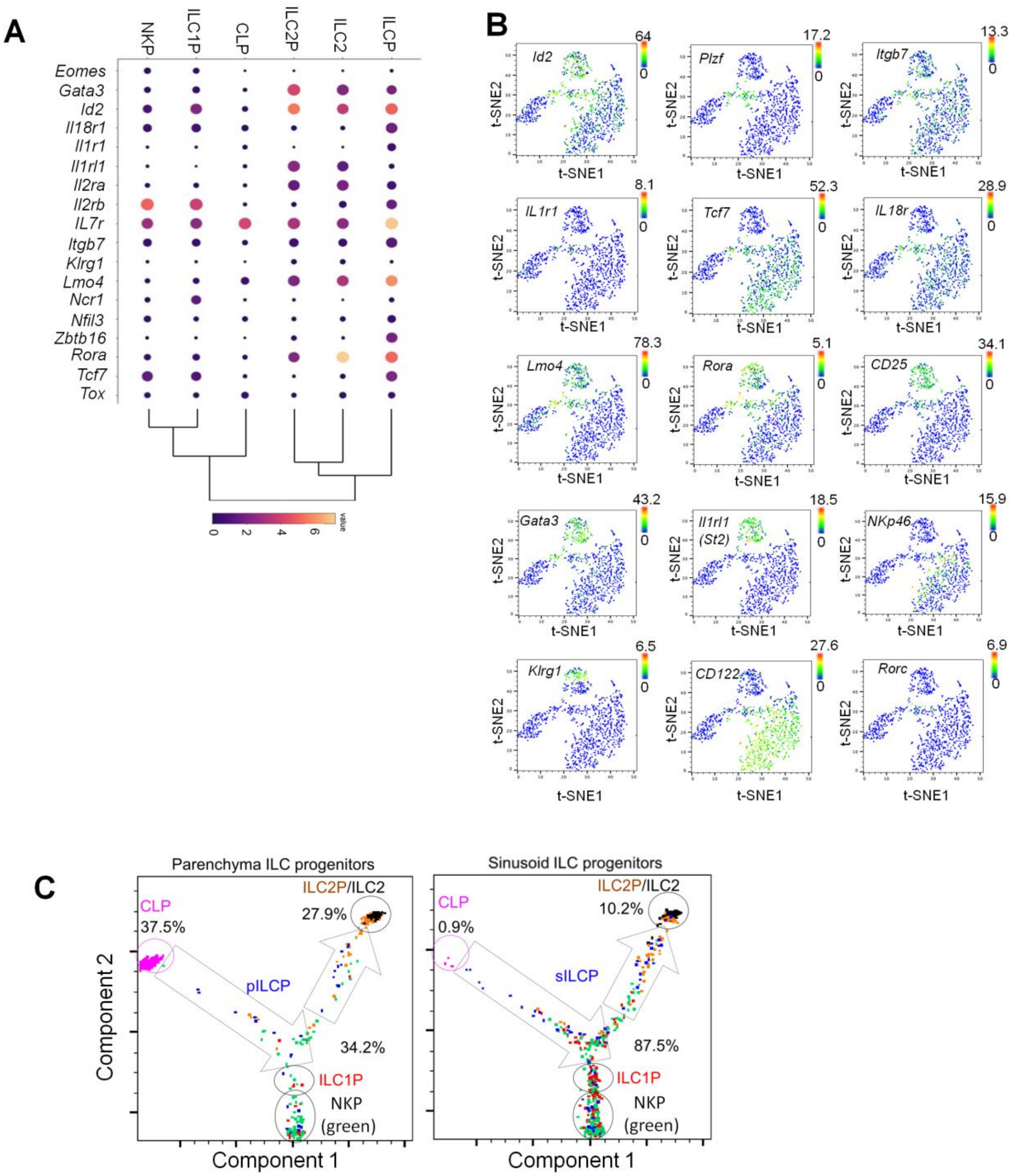

Supplementary Figure S4. Single cell transcriptome of BM parenchyma and sinusoid ILC progenitors. (A) Dot plot showing the expression of marker genes for various ILC progenitors. The color intensity of each circle represents average levels of gene expression (log scale), and the size of the circles represents the fraction of cells in each cluster in which transcripts for that gene was detected. (B) Feature t-SNE plot showing expression of signature marker genes on different subsets. (C) The Monocle pseudotime trajectory analysis was performed for BM parenchyma and sinusoid Lin$^-$ CD127$^+$ cells.

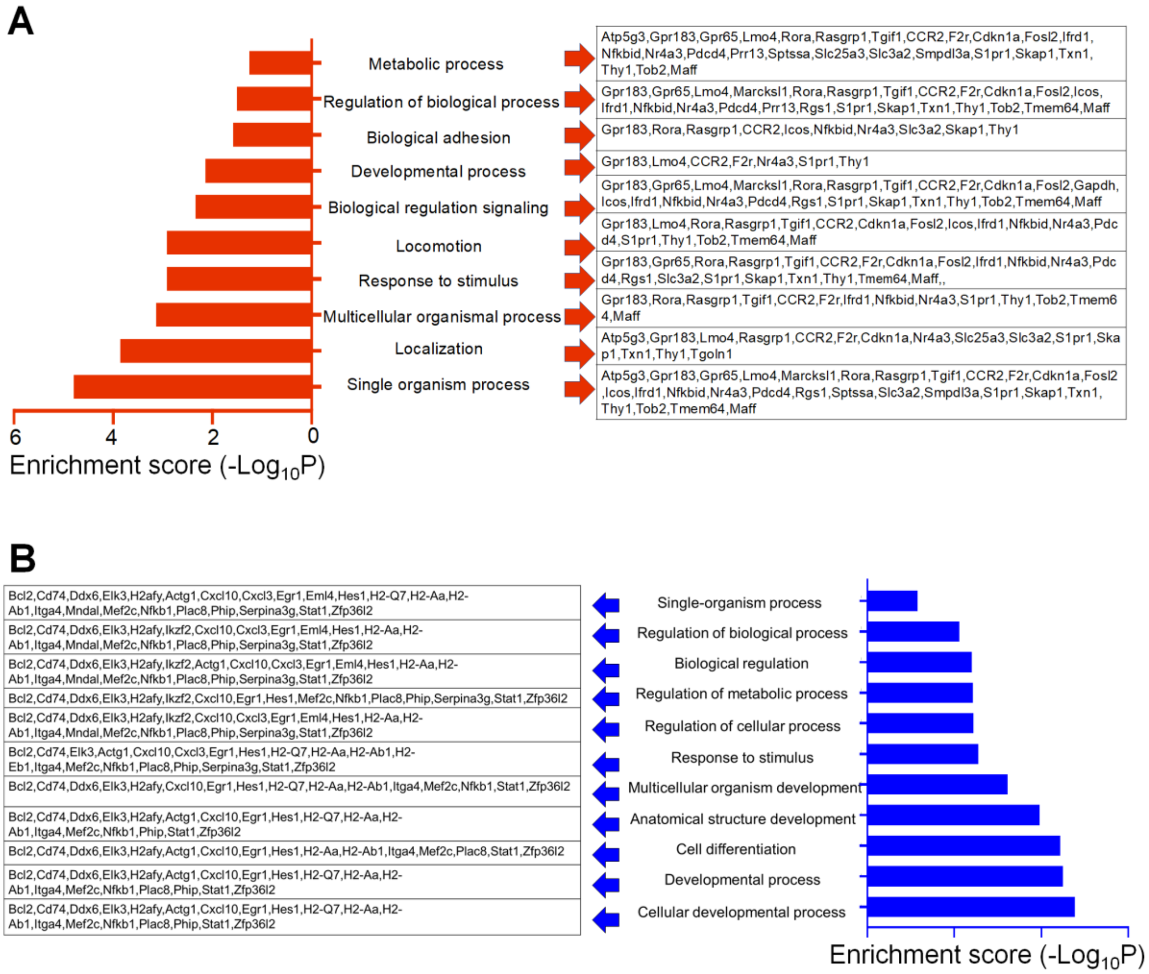

Supplementary Figure S5. Pathway analysis for the differentially regulated genes up-regulated in BM sILCP (A) and pILCP (B). KEGG (Kyoto Encyclopedia of Genes and Genomes) enrichment analysis for differentially regulated genes with DAVID (Database for Annotation, Visualization and Integrated Discovery) version 6.8 functional annotation tool (https://david.ncifcrf.gov/tools.jsp). Top pathways for the up-regulated genes in sILCP (A) and pILCP (B) are shown.

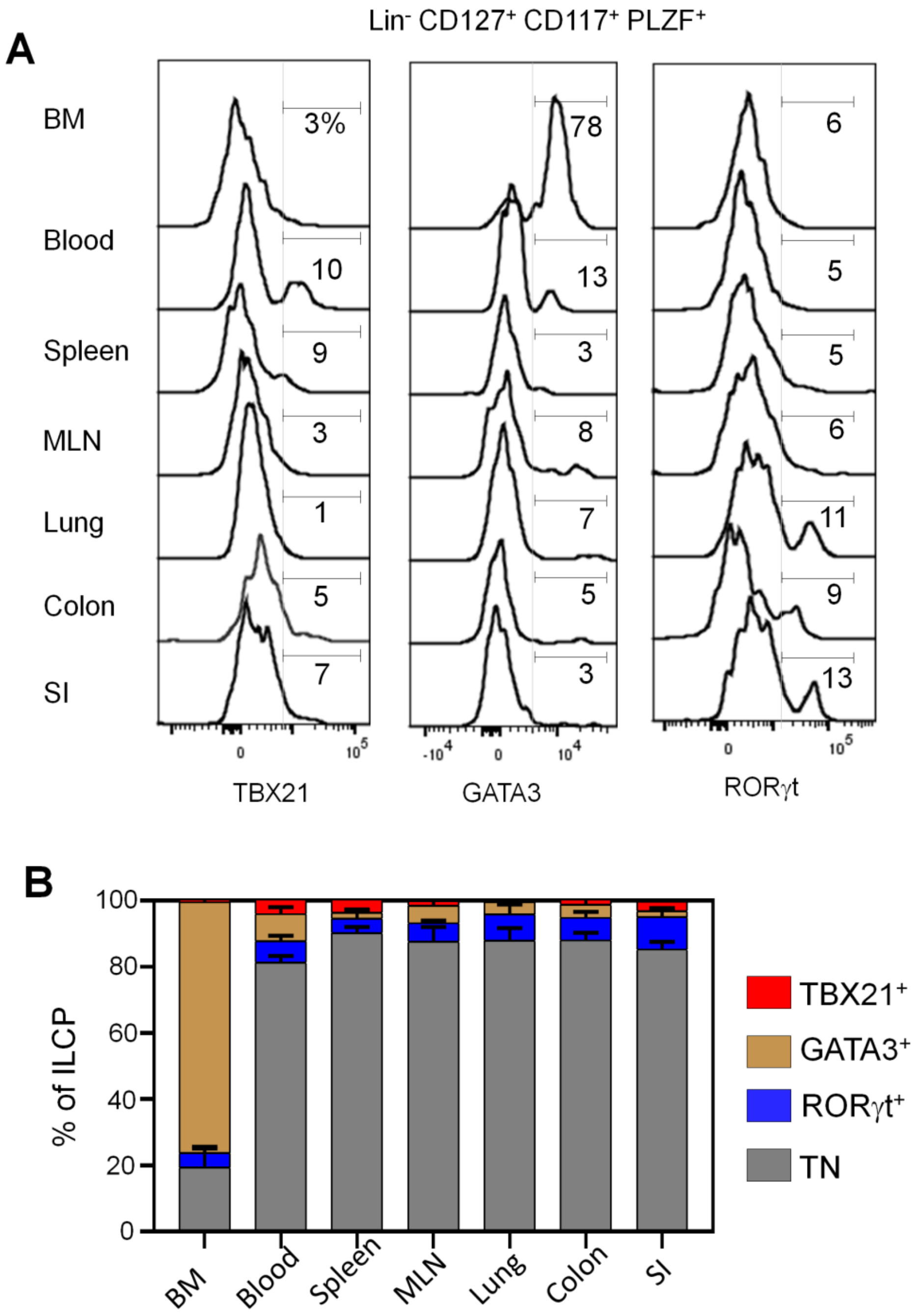

Supplementary Figure S6. The expression of TBX21, GATA3 and RORγt in Lin$^-$ CD127$^+$ CD117$^+$ PLZF$^+$ ILCP-like cells in the BM, peripheral blood, and indicated tissues. Representative and pooled data obtained from at least three different experiments are shown. Abbreviations for tissues: mesenteric lymph nodes (MLN) and small intestine (SI).

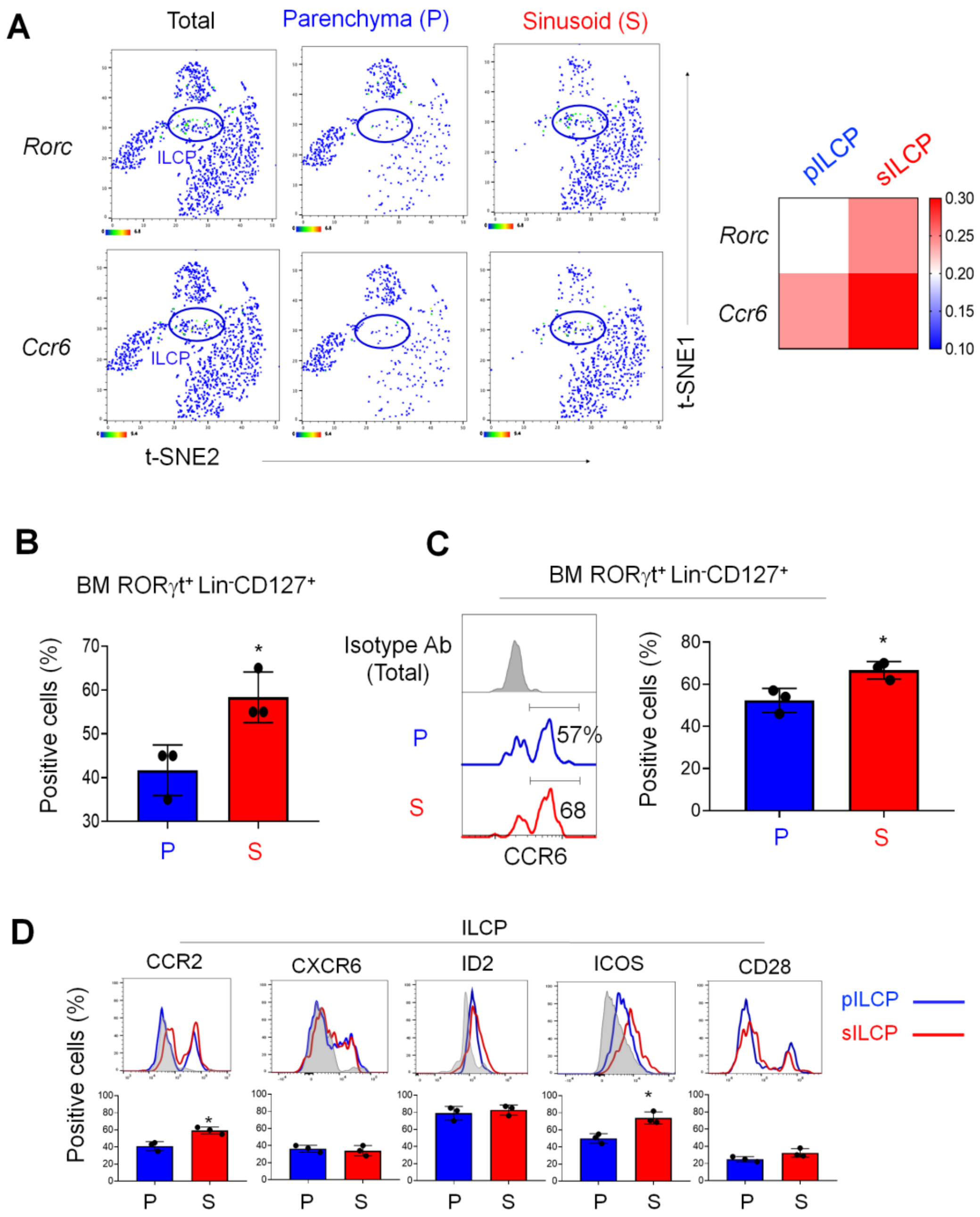

Supplementary Figure S7. The expression of RORγt and CCR6 by pILCP and sILCP. (A) Feature t-SNE plot and heat map showing expression of *Rorc* and *Ccr6* on pILCP and sILCP. (B) The relative distribution of BM RORγt$^+$CD127$^+$ cells in BM parenchyma vs. sinusoid niches. Sinusoid labeling with AmCyan-anti-CD45 was performed and the distribution of RORγt$^+$ ILCs in AmCyan-labeled and unlabeled fractions was determined by flow cytometry. (C) Expression of CCR6 by parenchyma vs. sinusoid RORγt$^+$CD127$^+$ cells. (D) Flow cytometry detection of selected molecules by sILCP and pILCP. Representative and pooled data obtained from at least three different experiments are shown. *Significant differences ($P < 0.05$).

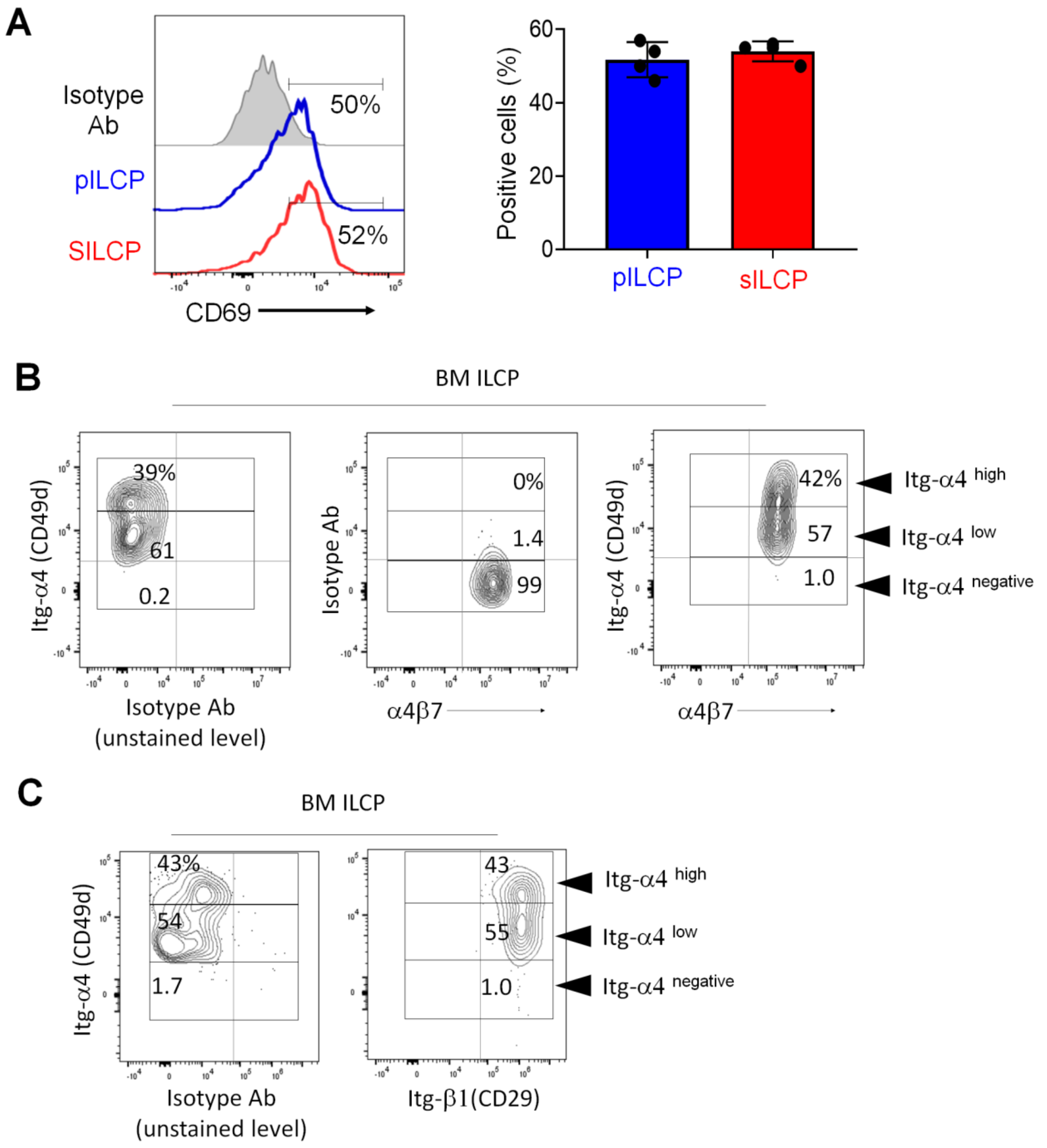

Supplementary Figure S8. The expression of CD69, Itg-α4, Itg-α4β7 and Itg-β1 by BM ILCPs. (A) The expression of CD69 on pILCP vs. sILCP. Flow cytometry detection of CD69 by sILCP and pILCP. Pooled data obtained from two independent experiments (n=4) are shown. **(B) Itg-α4 vs. Itg-α4β7 expression by BM ILCPs. (C) Itg-α4 vs. Itg-β1 expression by BM ILCPs.**

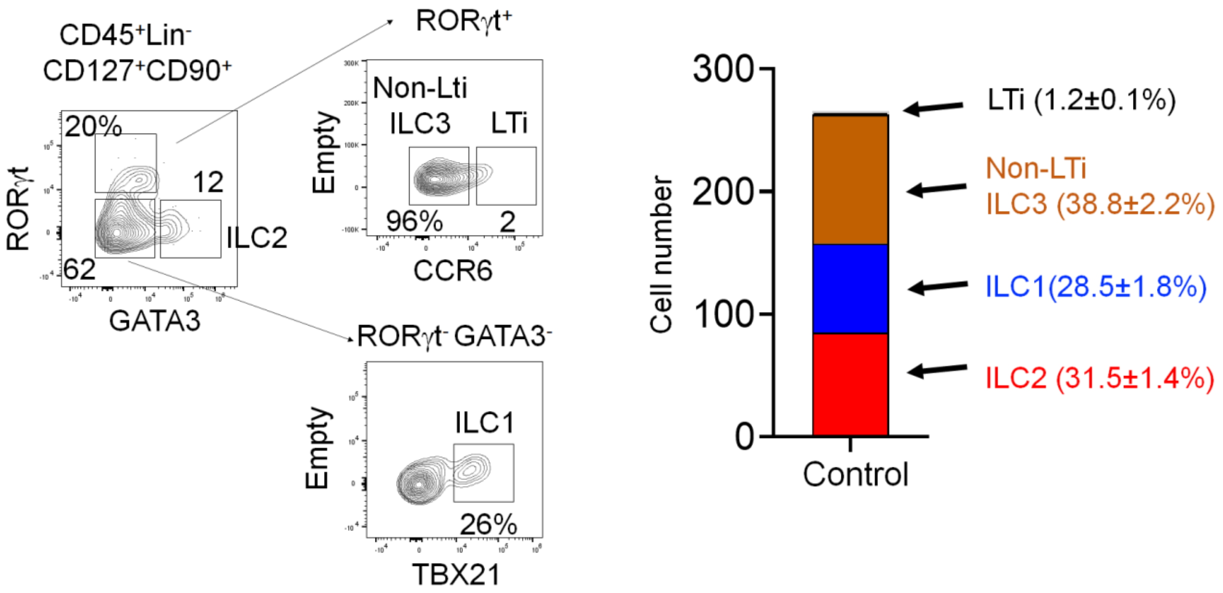

Supplementary Figure S9. The differentiation potential of BM sILCP. Sinusoid labeling with AmCyan-anti-CD45 was performed in Rag$^{-/-}$ mice and sorted BM sILCP were cultured on OP9-DL1 cells for 10 days. Indicated mature ILCs were examined after culture. Pooled data obtained from two independent experiments (n=4) are shown.

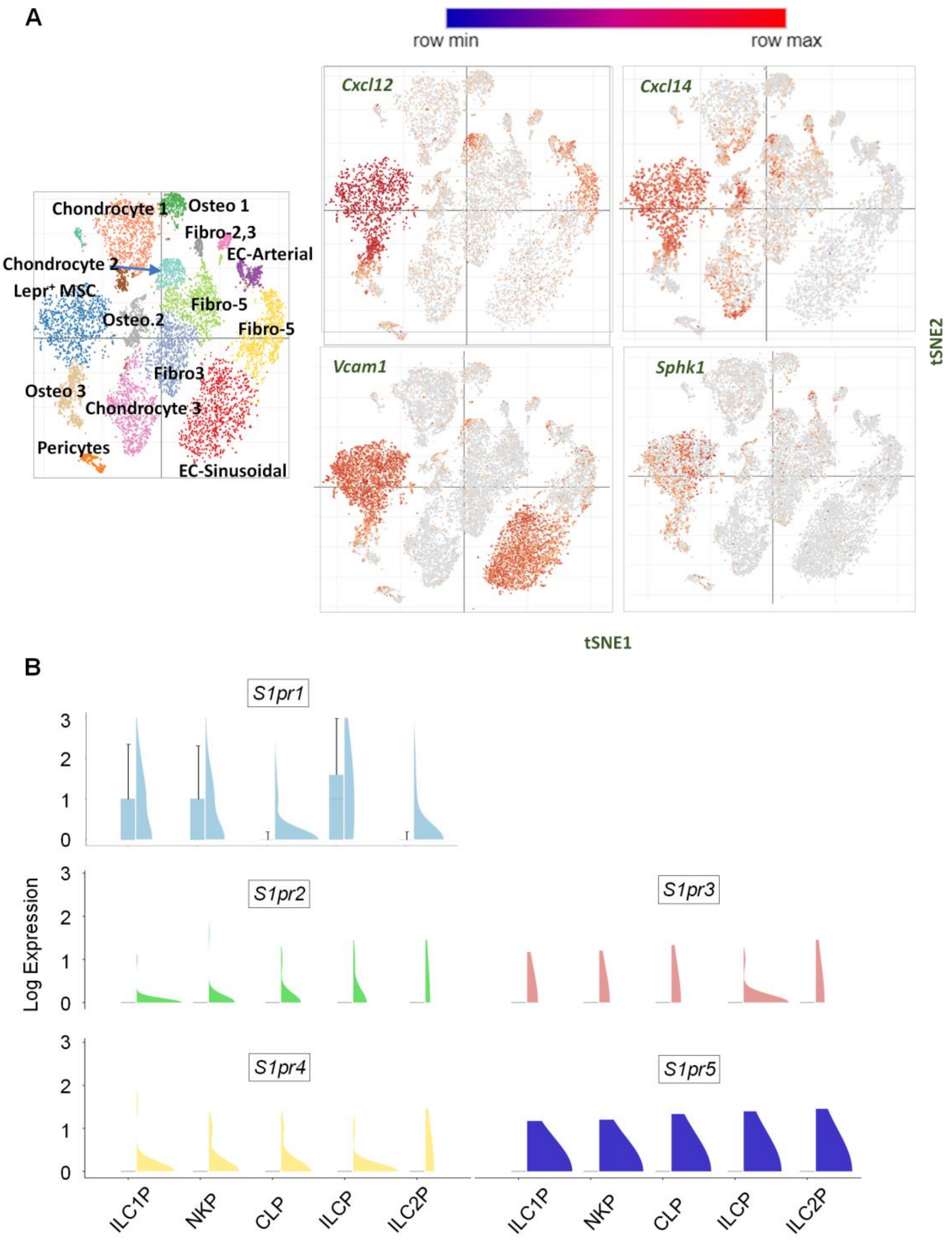

Supplementary Figure S10. The expression of trafficking signals by BM niche cells and the expression of S1P receptors by BM ILC progenitors. (A) Expression of the CXCR4 ligands (*Cxcl12* and *Cxcl14*), *Vcam1* and *Sphk1* in bone marrow niche cells such as mesenchymal stromal cells (MSC) and endothelial cells (ECs). The data were retrieved from publicly available scRNA-seq data (GSE128423). (B) Comparison of the expression of S1P receptor (S1PR1-5) at RNA level in the BM ILC progenitors. The data were from the scRNA-seq analysis performed in the current study.

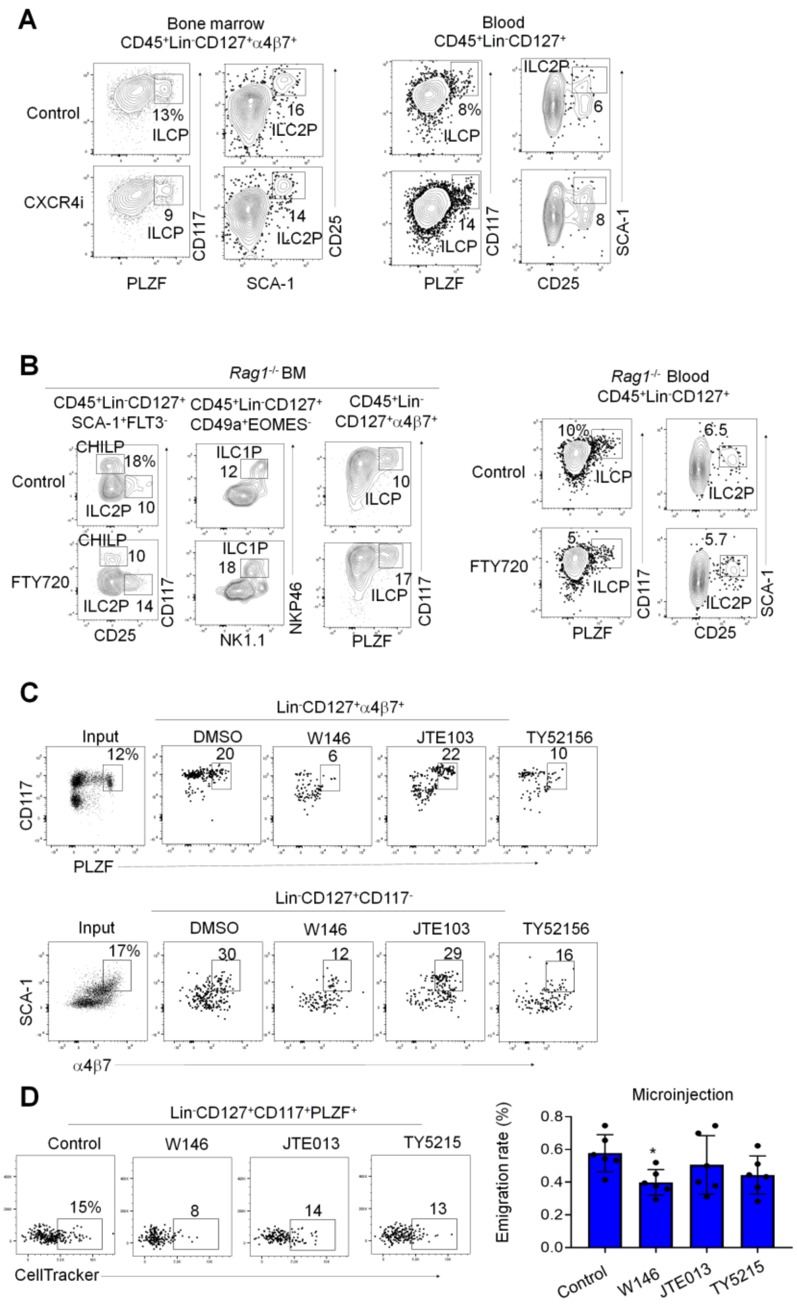

Supplementary Figure S11. The impact of CXCR4i and S1P receptor inhibitors on the mobilization of BM ILCPs. (A) Representative flow plots for the frequency of ILC progenitors in the BM and blood of Rag$^{-/-}$ mice treated with or without CXCR4i (AMD3100). (B) Representative flow plots for the frequency of ILC progenitors in the BM and blood of Rag$^{-/-}$ mice treated with or without FTY720. (C) Representative flow plots for the chemotaxis of BM ILCPs to S1P with or without the treatment with the S1pr1 antagonist W146, the S1PR2 antagonist JTE013, or the S1PR3 antagonist TY52156. (D) Emigration rates of BM ILCPs with or without the treatment with W146, JTE013, or TY52156. Pooled data obtained from at least three different experiments are shown. *Significant differences ($P < 0.05$).

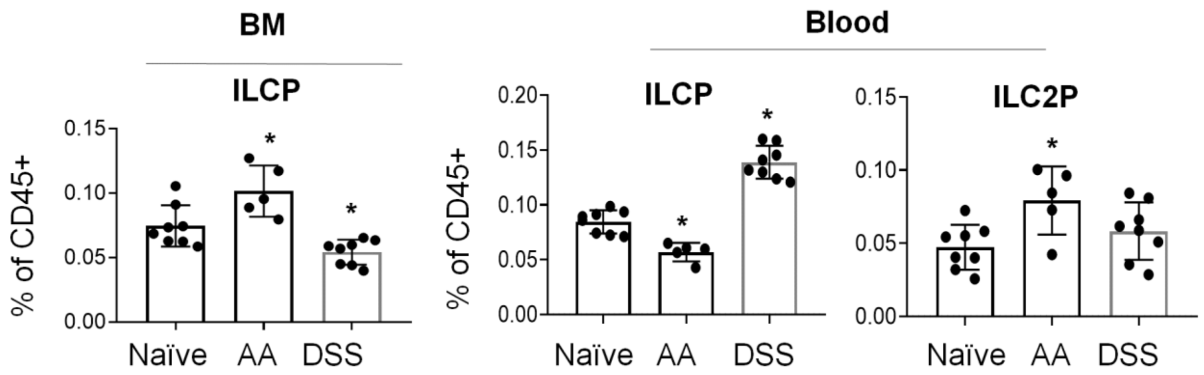

Supplementary Figure S12. Differential effects of DSS-induced gut and AA-induced lung inflammation on the frequencies of BM and blood ILCPs. The frequency of ILCPs in the BM and peripheral blood of mice challenged with *A. alternata* extract (AA, i.n.) or DSS in drinking water. Pooled data obtained from three independent experiments (n=5-8) are shown. *Significant differences (P < 0.05).

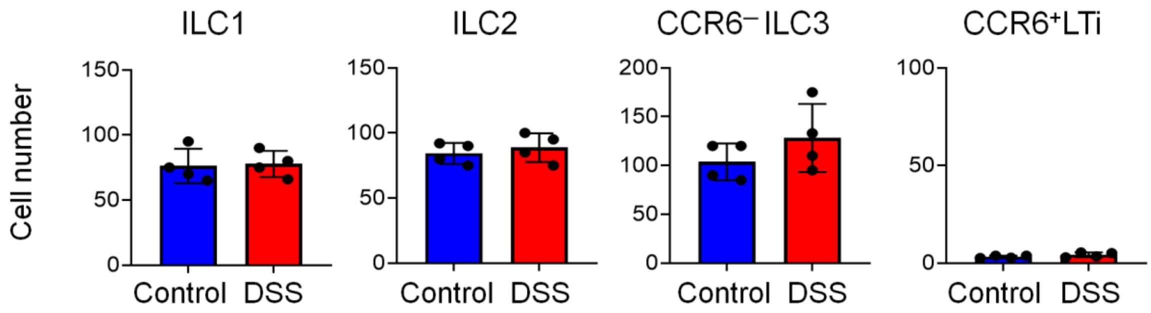

Supplementary Figure S13. Comparison of steady state and DSS-induced inflammation on the *in vitro* differentiation potential of BM sILCP. In vitro differentiation of BM sILCP from naïve vs. DSS-treated *Rag1*$^{-/-}$ mice. sILCP were sorted and cultured on OP9-DL1 cells for 10 days for flow cytometry. *Significant differences (n=4, $P < 0.05$).

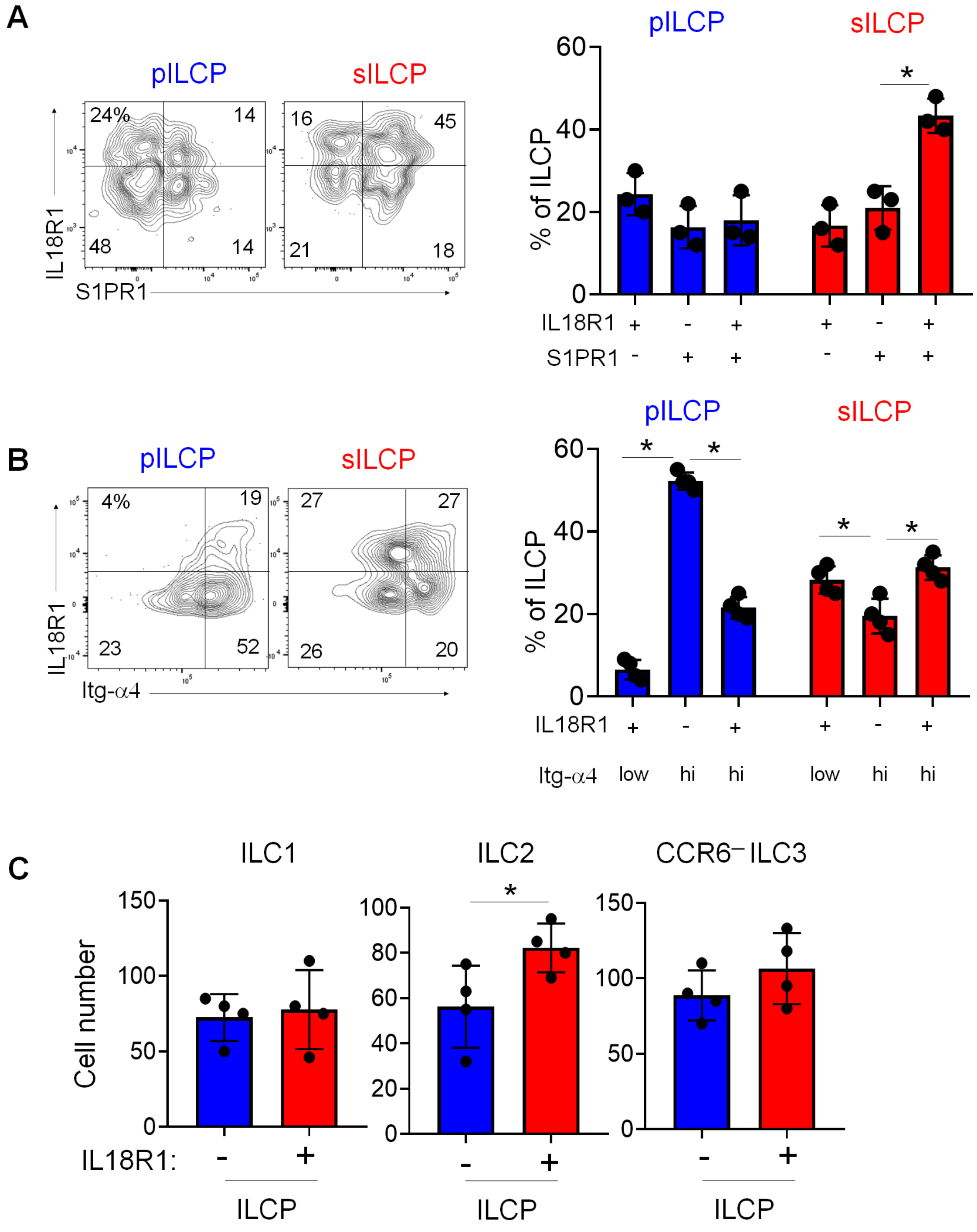

Supplementary Figure S14. Expression of S1PR1/Itg-α4 and differentiation potential of IL18R1^{+} and IL18R1^{-} ILCP subsets in the BM. (A) Expression of IL18R1 and S1PR1. (B) Expression of IL18R1 and Itg-α4. (C) *In vitro* differentiation of IL18R1^{+} vs. IL18R1^{-} BM ILCPs on OP9-DL1 cells for 10 days. Pooled data obtained from three different experiments are shown. *Significant differences (n=3-4).

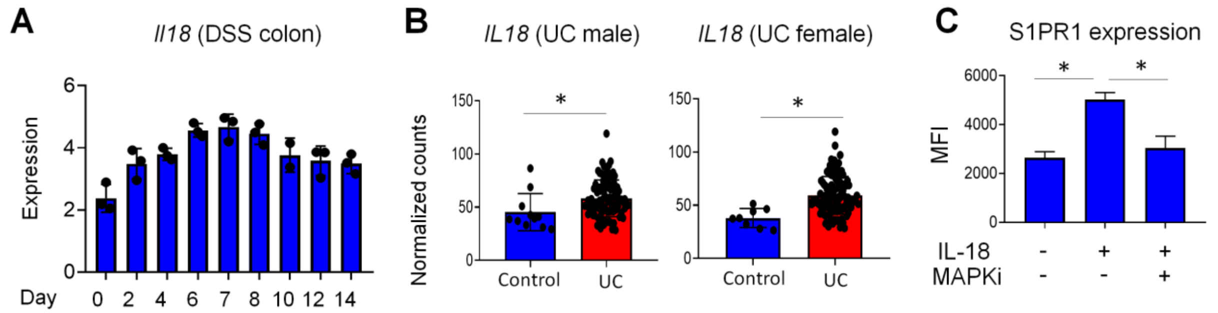

Supplementary Figure S15. Expression of IL-18 in inflamed colon tissues and the effect of MAPK inhibition on IL-18-induced S1pr1 expression. (A) The expression of IL-18 in the colon of control and DSS-treated mice. A publicly available RNA-seq data (GSE131032) was retrieved for IL-18 expression. (B) The expression of IL-18 in the colon of healthy and ulcerative colitis (UC) patients. A publicly available RNA-seq data (GSE109142) was retrieved for IL-18. (C) The effect of MAPK inhibition on IL-18-induced S1PR1 expression. *Significant differences (n=5, panel C).

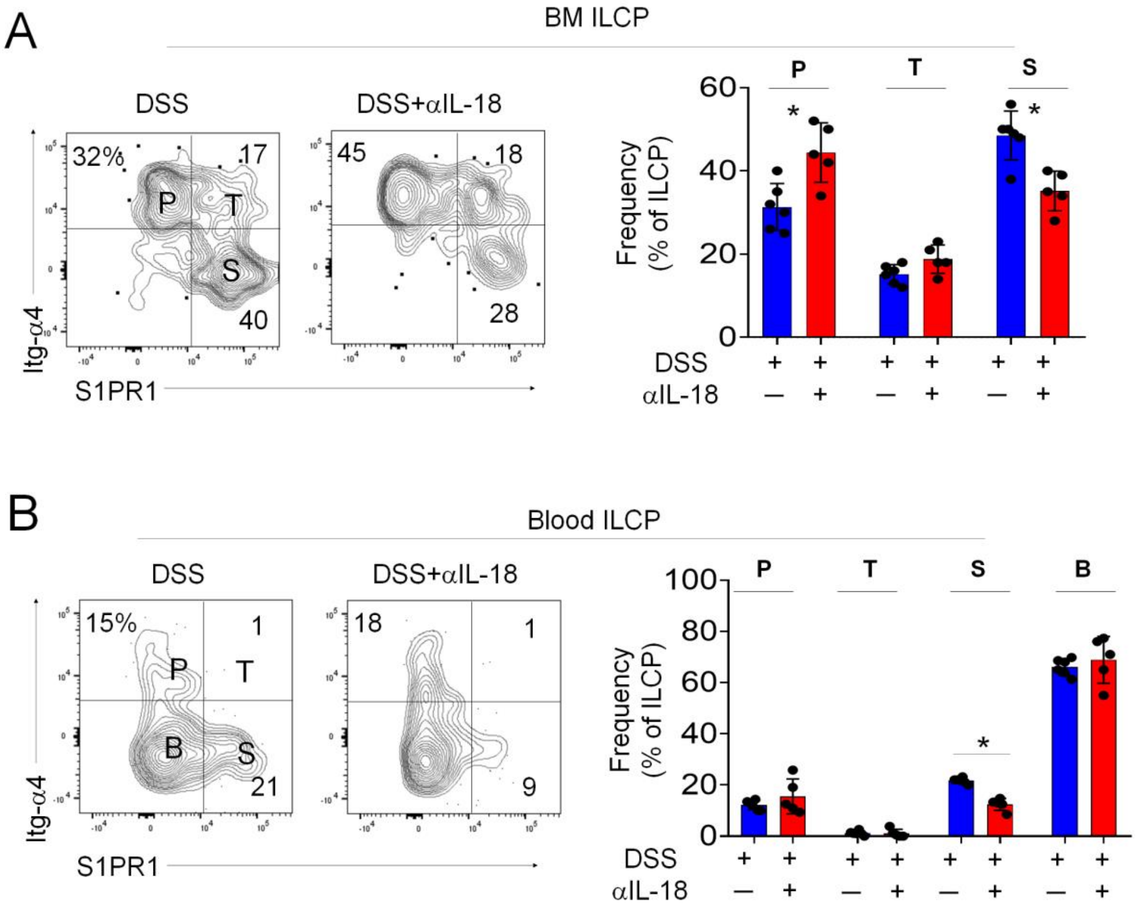

Supplementary Figure S16. Impact of IL-18 neutralization on the composition of BM and blood ILCP subsets defined by Itg-α4 and S1PR1. BM ILCPs defined by the expression of integrin α4 and/or S1PR1 were examined in the BM (A) and blood (B) of *Rag1*$^{-/-}$ mice treated with DSS with or without αIL-18 administration. Pooled data obtained from three different experiments are shown. *Significant differences (n=5).

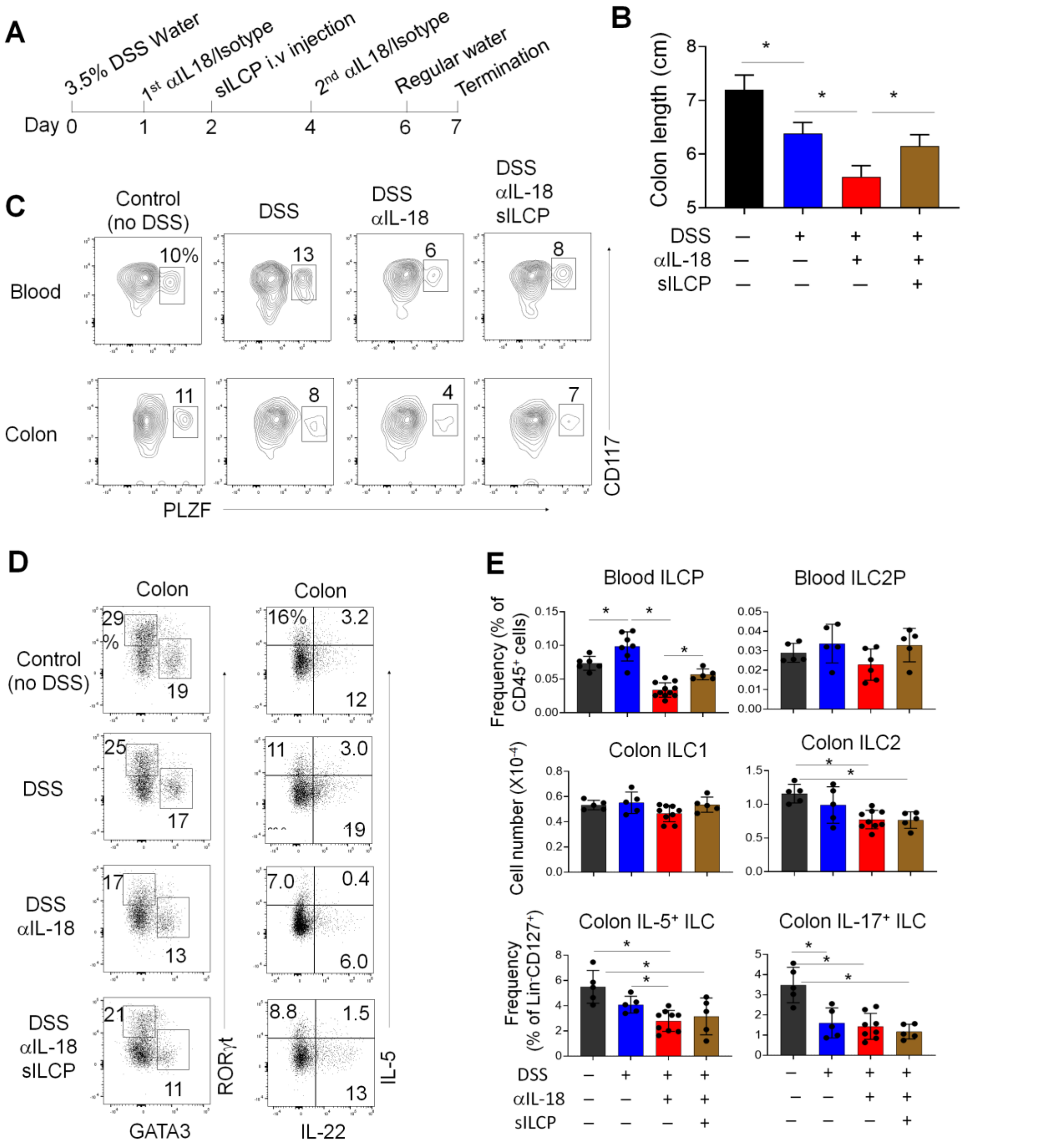

Supplementary Figure S17. The effect of infused sILCP on DSS-induced colitis and ILC activity. (A) The experimental flow for the administration of DSS in drinking water, neutralizing antibody (i.p.) and sILCP (i.v.). Colon length (B) and ILCP numbers (C) following sILCP transfer in *Rag1*$^{-/-}$ mice treated with aIL-18 and DSS to induce colitis. (D and E) Frequency and cytokine production by ILCs in the colon following sILCP transfer in *Rag1*$^{-/-}$ mice treated with DSS and/or αIL-18. Representative (C,D) and combined (E) data are shown. Pooled data obtained from at least three different experiments are shown. *Significant differences (n=5-8).

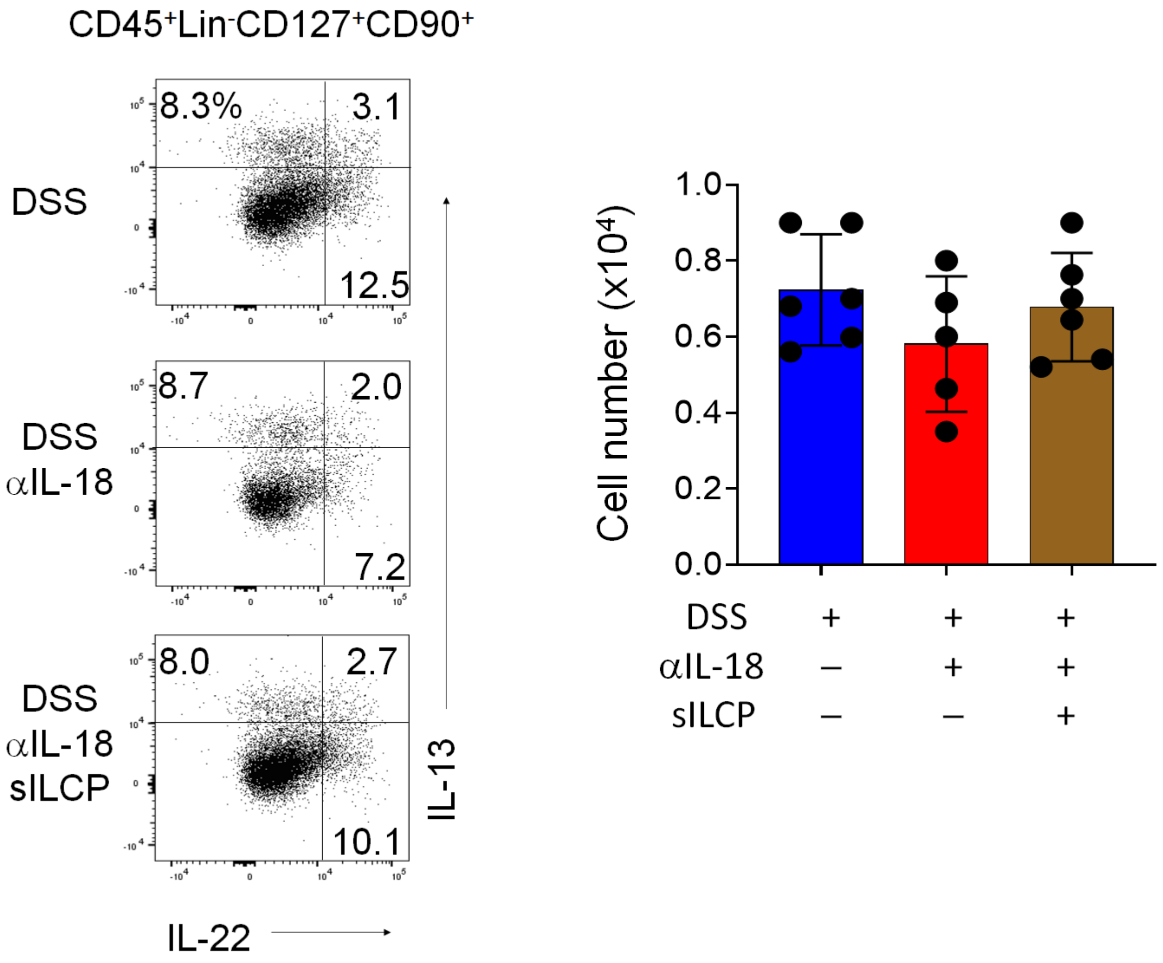

Supplementary Figure S18. The effect of infused sILCP on the number of IL-13^{+} ILCs in *Rag1*$^{-/-}$ mice treated with DSS and/or αIL-18. The treatment schedule is described in Supplementary Figure S17A. Colon ILCs were examined. *Significant differences (n=6).

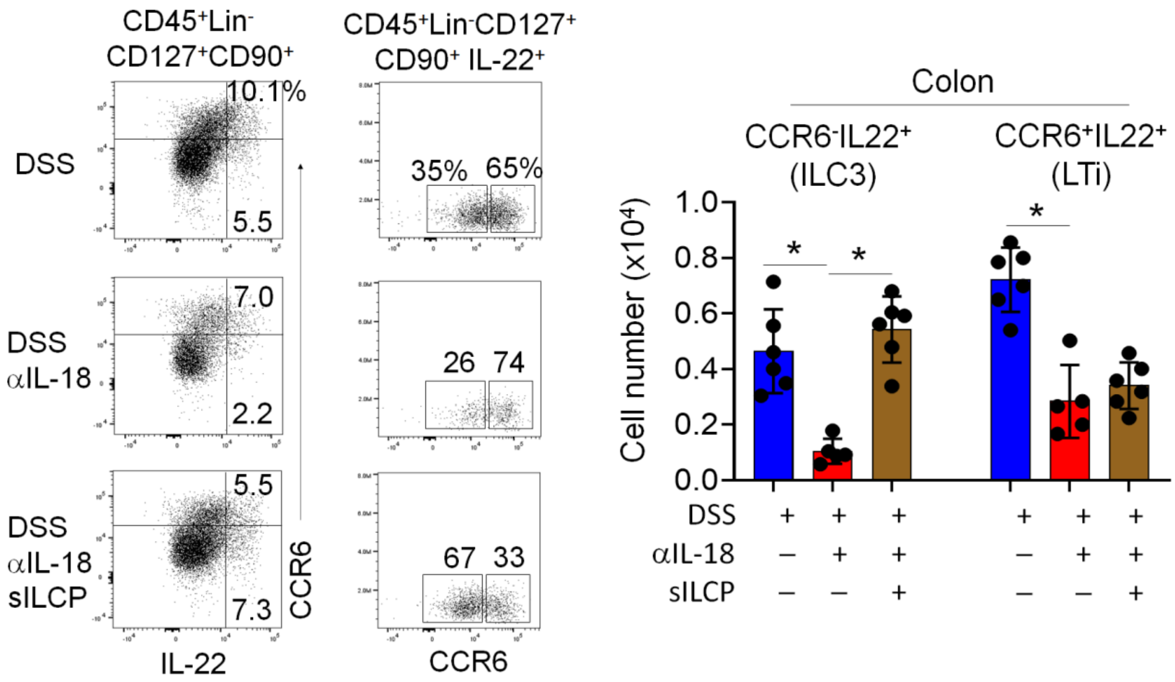

Supplementary Figure S19. The effect of infused sILCP on IL-22⁺ CCR6⁺ LTi vs. IL-22⁺ CCR6⁻ ILC3 cells in the colon following sILCP transfer in *Rag1⁻/⁻* mice treated with DSS and/or αIL-18. The treatment schedule is described in Supplementary Figure S17A. Colon ILCs were examined. *Significant differences (n=6 from 3 experiments).
